# Sporulation Strategies and Potential Role of the Exosporium in Survival and Persistence of *Clostridium botulinum*

**DOI:** 10.3390/ijms23020754

**Published:** 2022-01-11

**Authors:** Inês M. Portinha, François P. Douillard, Hannu Korkeala, Miia Lindström

**Affiliations:** Department of Food Hygiene and Environmental Health, Faculty of Veterinary Medicine, University of Helsinki, P.O. Box 66, 00014 Helsinki, Finland; ines.portinha@helsinki.fi (I.M.P.); francois.douillard@helsinki.fi (F.P.D.); hannu.korkeala@helsinki.fi (H.K.)

**Keywords:** *Clostridium botulinum*, spore, exosporium, morphology

## Abstract

*Clostridium botulinum* produces the botulinum neurotoxin that causes botulism, a rare but potentially lethal paralysis. Endospores play an important role in the survival, transmission, and pathogenesis of *C. botulinum*. *C. botulinum* strains are very diverse, both genetically and ecologically. Group I strains are terrestrial, mesophilic, and produce highly heat-resistant spores, while Group II strains can be terrestrial (type B) or aquatic (type E) and are generally psychrotrophic and produce spores of moderate heat resistance. Group III strains are either terrestrial or aquatic, mesophilic or slightly thermophilic, and the heat resistance properties of their spores are poorly characterized. Here, we analyzed the sporulation dynamics in population, spore morphology, and other spore properties of 10 *C. botulinum* strains belonging to Groups I–III. We propose two distinct sporulation strategies used by *C. botulinum* Groups I–III strains, report their spore properties, and suggest a putative role for the exosporium in conferring high heat resistance. Strains within each physiological group produced spores with similar characteristics, likely reflecting adaptation to respective environmental habitats. Our work provides new information on the spores and on the population and single-cell level strategies in the sporulation of *C. botulinum*.

## 1. Introduction

The endospore-forming, bacterial species *Clostridium botulinum* is divided into phylogenetically and ecologically distinct groups of clostridia that produce the highly potent botulinum neurotoxin (BoNT) [1]. The toxin blocks neurotransmission, causing a potentially lethal, flaccid paralysis known as botulism [2]. While BoNT is the causative agent of botulism, *C. botulinum* spores play a key role in the survival and dispersion of *C. botulinum* in the environment, in contamination of food and feed raw materials [3,4,5,6], and in pathogenesis [1,7]. Inadequate storage of contaminated food and feed products may result in spore germination and outgrowth into neurotoxinogenic *C. botulinum* cultures, posing a botulism risk for humans and animals [8,9,10]. Alternatively, spore germination in toxinogenic culture and colonization in the gut or in deep wounds may result in toxicoinfectious botulism [7,11]. Indeed, infant botulism with toxic gut colonization affecting babies under one year of age is the most common form of human botulism in the United States [12].

Sporulation has been extensively studied in the spore-forming model organisms *Bacillus subtilis* and *Clostridioides difficile* [13,14]. Sporulation is a stress response strategy to evade adverse environmental conditions, such as starvation or high cell density [15]. Upon initiation of sporulation, the asymmetrical septation leads to the formation of a mother cell and a forespore [13]. The forespore is then engulfed and surrounded by protective layers by the enveloping mother cell. These layers determine the spore properties, resistance, and how it interacts with its environment [16]. Earlier spore research in *C. botulinum* focused on the regulation and activation of sporulation [17,18,19,20], spore heat resistance [9,21,22], and germination [23]. Sporulation triggers, the proportion of sporulating cells, and the timeline of spore maturation and release can vary depending on the strain or species and the environmental conditions. In laboratory conditions, *B. subtilis* strains, for example, have similar sporulation strategies with the same sporulation trigger, similar proportions of sporulating cells, and similar sporulation times, while sporulation strategies of *C. difficile* vary by strain and medium [15,24,25].

The sporulation strategies of *C. botulinum* are poorly described, but differences between spores of Group I and II strains are evident [26]. Dormancy and resistance to physical and chemical stressors allow spores to survive harsh environmental conditions, such as high temperature, aerobiosis, desiccation, and UV radiation [16], that would kill or damage vegetative cells. Resistance to high temperatures is a relevant aspect of *C. botulinum* spores as it allows the spores to resist common food and feed processing treatments. Inactivation of spores of the proteolytic Group I strains requires autoclaving or sterilization treatments, while spores from the non-proteolytic Group II strains can be destroyed by pasteurization [22]. Spores from Group III strains that can be either proteolytic or non-proteolytic present a variable heat resistance, usually higher than that of Group II strains and lower than that of Group I strains [22].

All spores have a basic structure composed of a dehydrated core, a peptidoglycan cortex, and a multilayer protein coat. However, some bacterial species or strains produce an additional structure called exosporium [16]. The exosporium shows substantial morphological differences between clostridia and bacilli species [27,28,29,30,31]. Thus, in *Bacillus cereus* and *Bacillus anthracis*, the exosporium is thin, loose, and possesses external hair-like projections [27]. Spores of *C. difficile* usually present a thick exosporium that is tightly attached to the coat and has hair-like fibrils, whereas in *Clostridium sordellii*, the exosporium presents a smooth, balloon-like structure [27,28]. Early *C. botulinum* studies showed a variety of spore morphotypes—thick and loose exosporium, antennae-like structure protruding from the coat surrounded by a thin and frail exosporium, and no exosporium [29,30,31]. Spores are able to aggregate and to adhere to organic and inorganic surfaces and to host cells, which, for some bacteria, is an integral part of their survival and infection strategies [32,33]. The spore adherence properties can relate to their hydrophobicity and their ability to autoaggregate, and these can be mediated by the exosporium. Hydrophobic bonds allow the spore to interact with other particles or surfaces and promote active adherence [33,34]. Autoaggregation is another trait associated with pathogenic adhesion and host colonization [35,36]. In *B. anthracis*, the hair-like projections are essential in spore adherence to top soil, allowing them to remain accessible to grazing ruminants, and are also needed for germination in the host organism [27,37]. In *B. cereus*, the exosporium mediates adhesion to inert food preparation surfaces [38]. In *C. difficile*, the exosporium is involved in adherence to inert surfaces, pathogenesis, and germination [39,40,41]. Currently, little is known of the functional properties of the different spore morphotypes in *C. botulinum*.

Here, we analyzed the sporulation diversity of 10 *C. botulinum* Group I, II, or III strains by specifically looking at (i) dynamics in the population structure in sporulating cultures and (ii) the ultrastructure and (iii) functional properties of individual spores. Remarkably, we report two distinct population dynamic patterns in sporulation, which could relate to the proteolytic properties of the strains of different Groups. Group I strains showed higher proportions of sporulating cells but took longer to release spores, ultimately resulting in higher spore counts than Group II and III strains. We identified four different spore morphotypes and found that most *C. botulinum* spores are highly hydrophobic and, in some cases, display active spore autoaggregation. Finally, strains with the lowest heat resistance within their respective group either lacked an exosporium or had a thinner exosporium compared to their phylogenetically related counterparts, suggesting a possible role of the exosporium in heat resistance.

## 2. Results

### 2.1. Sporulation Dynamics

Active cultures of all *C. botulinum* Group I and III strains and strain Beluga of Group II initiated sporulation within the first 24 h after inoculation, while Group II strains CB11/1-1 and Eklund 17B initiated sporulation after 48 h (Table 1, images in Figure S1).

Sporulation progressed slowly for Group I strains, and we observed slow accumulation of sporulating cells and only a small number of free spores at the end of the monitoring period. In Group II and III strains, sporulation progressed faster than in Group I strains as they did not display accumulation of sporulating cells but had a dramatic increase in the proportion of free spores (Table 1). However, two Group II strains had low cell density at the assumed mid-logarithmic time point and yielded no cell pellet. Some free spores were phase-dark, indicating rehydration of their cores either due to damage or to germination. In Group II and III strains, we observed free phase-dark spores appearing as early as 48 h and accumulating in these cultures. This cell type was only detected in one strain from Group I, ATCC17841, and in a very low percentage (0.3 ± 0.2%, Table 1).

### 2.2. Ultrastructure of Sporulating Cells and Spores

**Figure 1:**
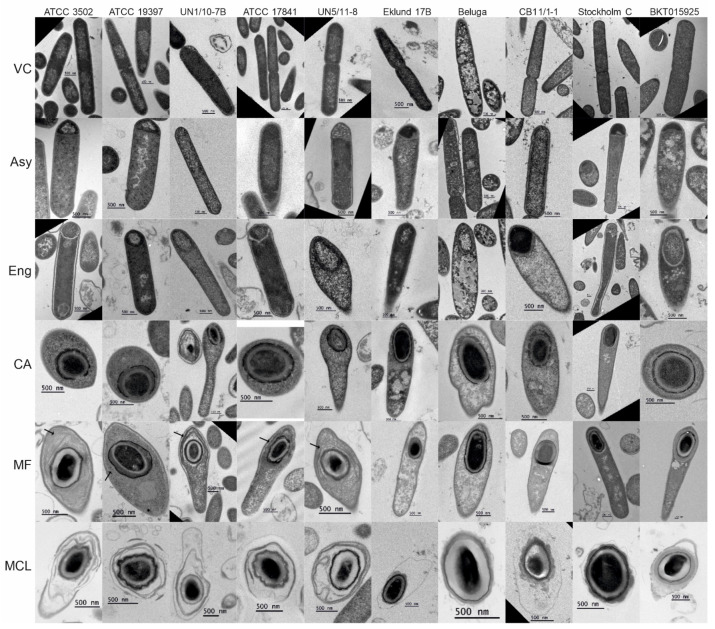
Electron micrographs of sporulating *Clostridium botulinum* cells. Images of thin sections of fixed, sporulating *C. botulinum* cultures were acquired to observe the ultrastructure of sporulating cells. The cells were categorized according to their morphological stages: VC, vegetative cell; Asy, asymmetric division; Eng, engulfment; CA, coat assembly; MF, mature forespore; MCL, mother cell lysis. Exosporia visible while the forespore was still in the mother cell are indicated with black arrows. Scale bars represent 500 nm. Enlarged versions of these images can be found in Figures S2 and S3.

We used transmission electron microscopy to analyze the ultrastructure of cells in different stages of sporulation and spores in *C. botulinum* Group I–III cultures (Figure 1). In all strains, we observed asymmetric septum formation and full engulfment of the forespore before the formation of the coat (Figure 1, Asy and Eng panels), unlike the earlier coat formation in *Clostridium tetani* and *Clostridium histolyticum* [42].

**Figure 2:**
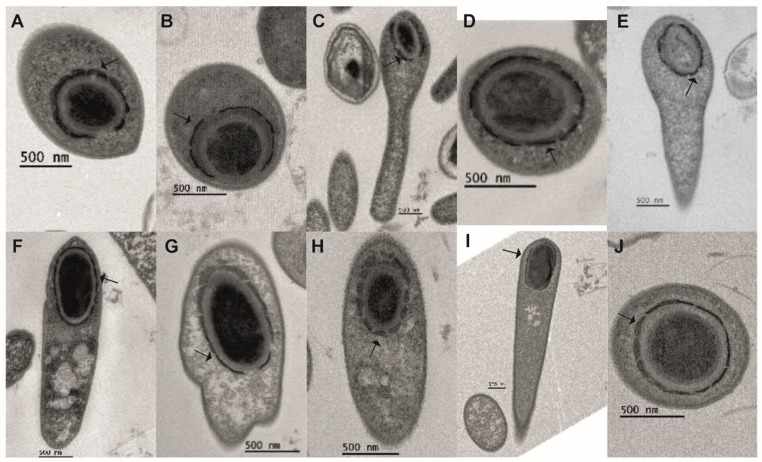
Electron micrographs depicting the coat assembly in sporulating *Clostridium botulinum* cells. (**A**) ATCC 3502, (**B**) ATCC 19397, (**C**) UN1/10-7B, (**D**) ATCC 17841, (**E**) UN5/11-8, (**F**) Eklund 17B, (**G**) Beluga, (**H**) CB11/1-1, (**I**) Stockholm C, (**J**) BKT015925. Coat fragments are indicated with black arrows.

In all 10 strains, the coat was assembled from small subunits that surrounded the forespore (Figure 2) and, apparently, fused together, forming a mature coat. In cells of Group I strains, an exosporium surrounding the forespore was visible while still in the mother cell (Figure 1, MF panel, black arrows).

**Figure 3:**
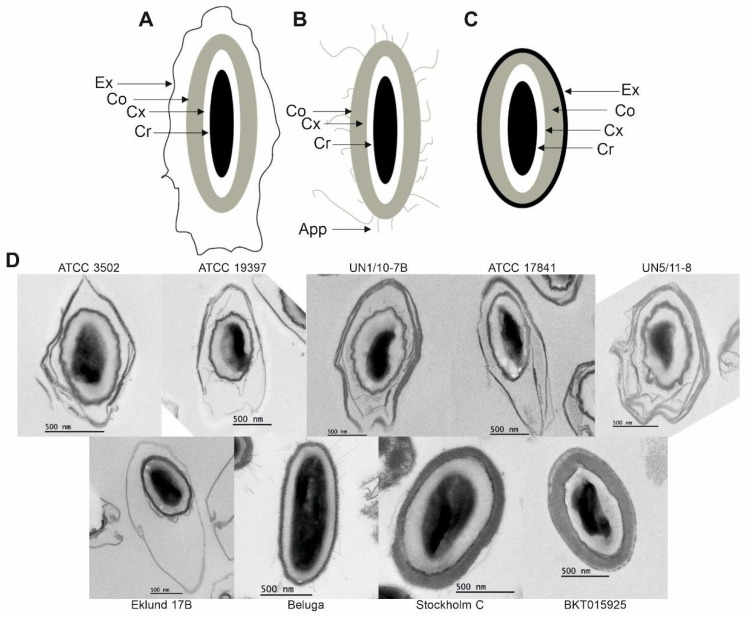
Morphology of purified *Clostridium botulinum* spores. (**A**) Typical morphologies of Group I strains and Group II Eklund 17B, (**B**) Group II Beluga, and (**C**) Group III strains, presenting: Cr, core; Cx, cortex; Co, coat; Exp, exosporium; and App, appendages. (**D**) Electron micrographs of thin sections of purified spores from ATCC 3502, ATCC 19397, UN1/10-7B, ATCC 17841, UN5/11-8, Eklund 17B, Beluga, Stockholm C, and BKT015925.

**Figure 4:**
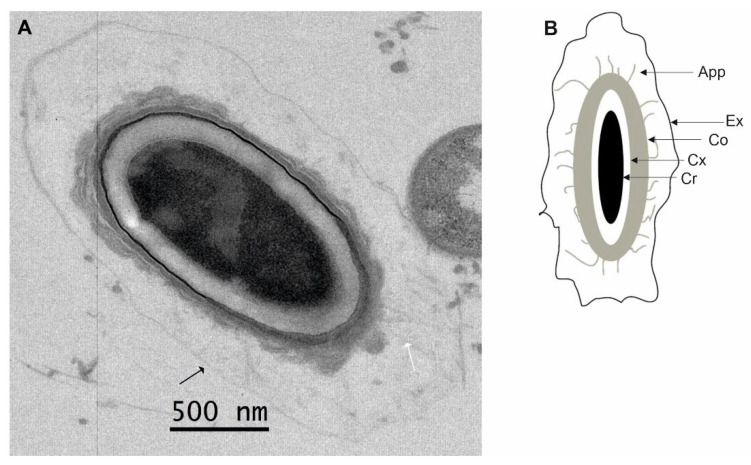
Morphology of non-purified spores of *Clostridium botulinum* CB11/1-1. CB11/1-1 spores show a morphology reminiscent of both Beluga and Eklund 17B morphologies. (**A**) CB11/1-1 spore displaying both appendages (white arrow) and exosporium (black arrow). (**B**) The morphologic scheme of CB11/1-1 spore: Cr, core; Cx, cortex; Co, coat; Exp, exosporium; and App, appendages.

Spores of both Group III strains and those of Group II CB11/1-1 and Eklund 17B also harbored an exosporium (Figure 3 and Figure 4); however, this structure was very thin and was not visible in cells carrying a mature spore (Figure 1, MF panel). Both free and purified spores of Group II Beluga did not present an exosporium (Figure 1 and Figure 3). Three spore morphotypes were identified based on TEM images of purified spores (Figure 3). All Group I strains and Group II Eklund 17B displayed spores surrounded by a loose-fitting exosporium with a lamellar structure (Figure 3A,D). Spores of both Group I isolates from botulism cases UN1/10-7B and UN5/11-8 appeared to show a multi-layered exosporium. Group II Beluga spores, instead, presented with antenna-like appendages and no exosporia at all (Figure 3B,D). Spores of both Group III strains showed thin, tight-fitting exosporia (Figure 3C,D).

Unfortunately, we were not able to purify CB11/1-1 spores due to the presence of excess cell debris and free phase-dark spores. However, free CB11/1-1 spores found in sporulating cultures in TEM images displayed a fourth morphotype with appendages typical for Beluga spores and an exosporium typical for Eklund 17B spores (Figure 4). Free spores of Beluga and Eklund 17B did not present this morphotype (Figure 1, MCL panel).

A systematic analysis of the structures of purified spores of each strain was performed by taking measurements of 13 key spore structures, as observed in the TEM images of 10 individual spores of each strain (Tables S1 and S4).

Only spores that appeared to be centrally cut by their longest axis were selected for this analysis in order to measure their longest and widest points and the correct thickness of their structures. The absence and poor visibility of Beluga, BKT015925, and Stockholm C exosporia removed their eligibility for related measurements.

Overall, when considering the exosporium, Group I strains and Group II Eklund 17B had the longest spores, and the Group I strains UN1/10-7B and UN5/11-8 and Group II Eklund 17B had the widest spores (Figure S4A,B).

Excluding the exosporium, Group II Beluga and Group III Stockholm C had the longest spores (Figure S4C), and Group III Stockholm C had the widest spores. Group I UN1/10-7B and UN5/11-8 and Group II strains Beluga and Eklund 17B had medium-wide spores, and Group I strains ATCC 3502, ATCC 19397, and ATCC 17841 had the thinnest spores (Figure S4D).

**Figure 5:**
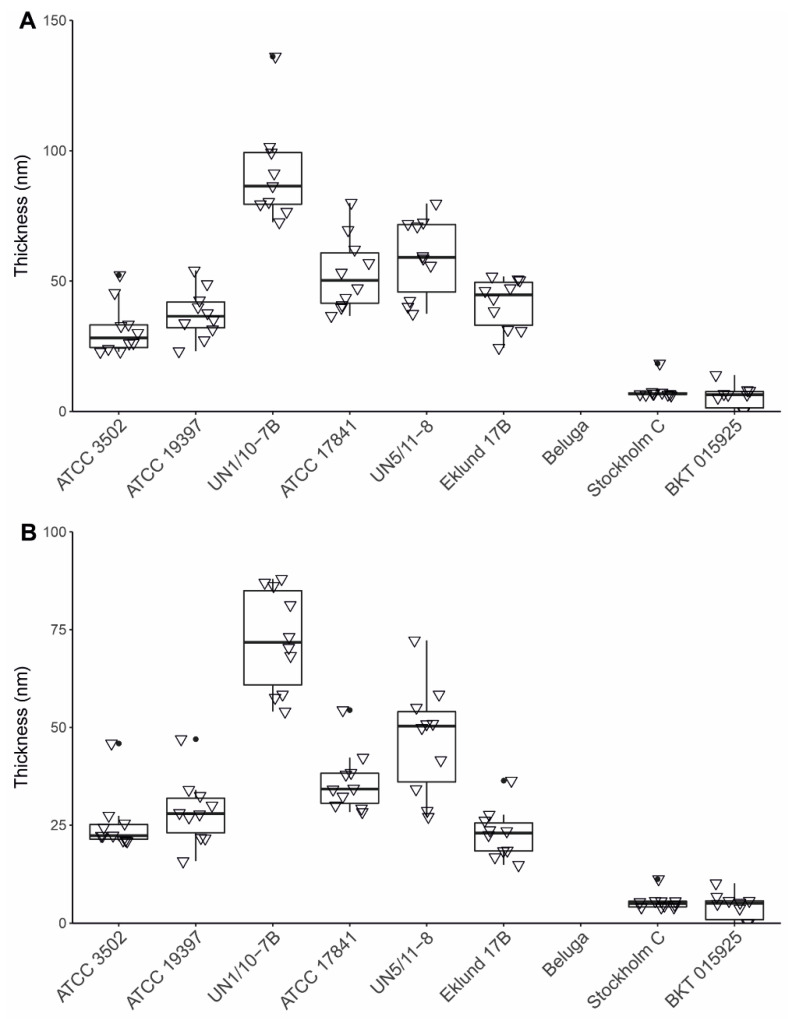
Thickness of the exosporium in *Clostridium botulinum* spores. The exosporium thickness of 10 spores of each purifiable strain was measured in three projections per spore. (**A**) The average thickness and (**B**) the minimum measured thickness of the exosporia were plotted in a scatter plot and boxplot combination to show both data density and distribution. UN1/10-7B was the only strain significantly different from others (*p* < 0.01).

The Group III strains had the thickest coats (Figure S4H). We analyzed the exosporia and their position relative to the coats. Group I strains UN1/10-7B and UN5/11-8 and Group II Eklund 17B had the largest space between the coat and the exosporium, while Group III strains had the smallest (Figure S4I). The spacing from the top of the coat to the exosporium and bottom of the coat to the exosporium were consistent across the strains (Figure S4J,K). Group I UN1/10-7B had a thicker exosporium than any other strain (Figure 5A,B and Figure S4L).

Finally, the only strain to present appendages was Beluga. The number of appendages per spore varied in the observed spores from none to nine, and their length varied from 47 to 642 nm (Figure S4M).

### 2.3. Spore Autoaggregation, Sedimentation, and Hydrophobicity

**Figure 6:**
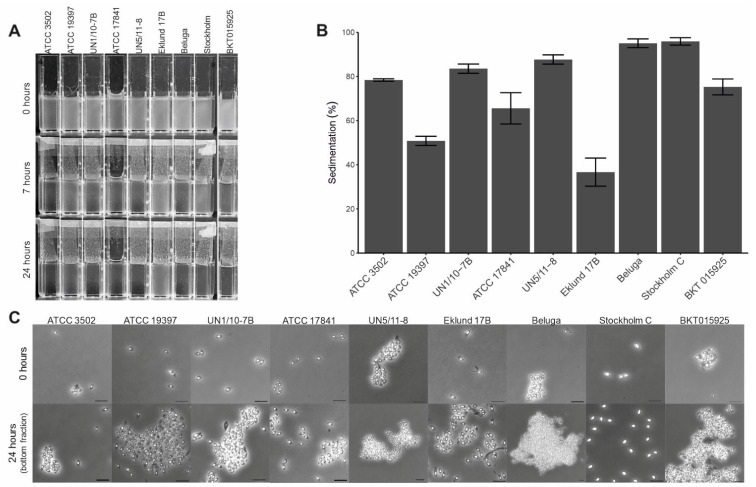
Autoaggregation of *Clostridium botulinum* spores. (**A**) Photographic screening of the rate of sedimentation in spore suspensions of each strain, (**B**) assessment of spore deposition by decrease in OD_600nm_, columns representing the average of three replicates with standard deviation shown by the error bars. (**C**) Phase contrast microscopy of the initial suspension and bottom fraction after 24 h of deposition. Scale bars represent 2 µm.

We tested spore autoaggregation by allowing the spore suspensions to sediment. While all spore suspensions eventually sedimented at the bottom of the cuvette, spores capable of autoaggregating were expected to attach to each other and sedimented faster. Active autoaggregation was observed in Group I strains UN1/10-7B and UN5/11-8, Group II Beluga, and Group III BKT015925. Spore suspensions from these strains sedimented in less than 6 to 7 h and had high sedimentation values from 96% to 84% (Figure 6A,B). Using phase contrast microscopy, we observed autoaggregation in these strains (Figure 6C). Other strains did not display autoaggregation. Instead, their spore suspensions displayed passive sedimentation with long periods to clear the suspensions (Figure 6A), lower sedimentation values (78% to 51%, 37% for Eklund 17B—Figure 6B), and an absence of autoaggregation (Figure 6C). 

**Figure 7:**
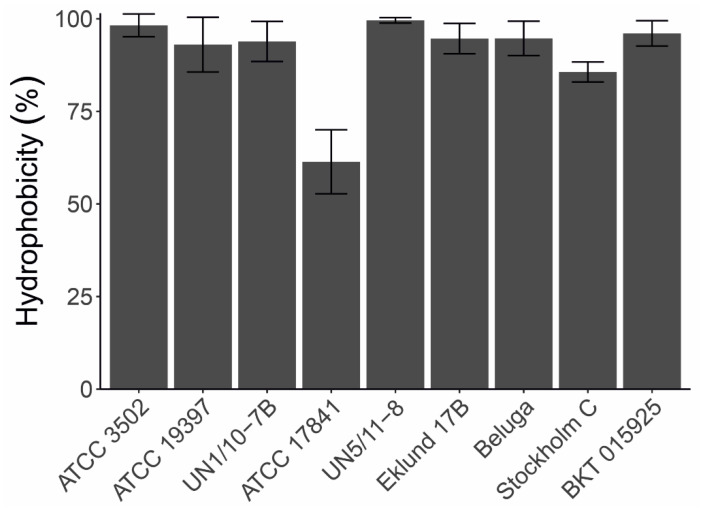
Hydrophobicity of *Clostridium botulinum* spores. The capacity of the purified spores of each strain to adhere to hexadecane was tested in a BATH assay; bars represent the average of three technical replicates with standard deviation shown by the error bars.

We studied the hydrophobicity of *C. botulinum* spores with the BATH assay [33], measuring the adhesion of purified spores to hexadecane by monitoring the decrease of OD_440nm_ in aqueous solution. Most strains had hydrophobic spores with hydrophobicity values varying from 86% to 100%, with the only outlier being ATCC 17841 (61%, Figure 7).

### 2.4. Thermal Destruction of Spores

**Figure 8:**
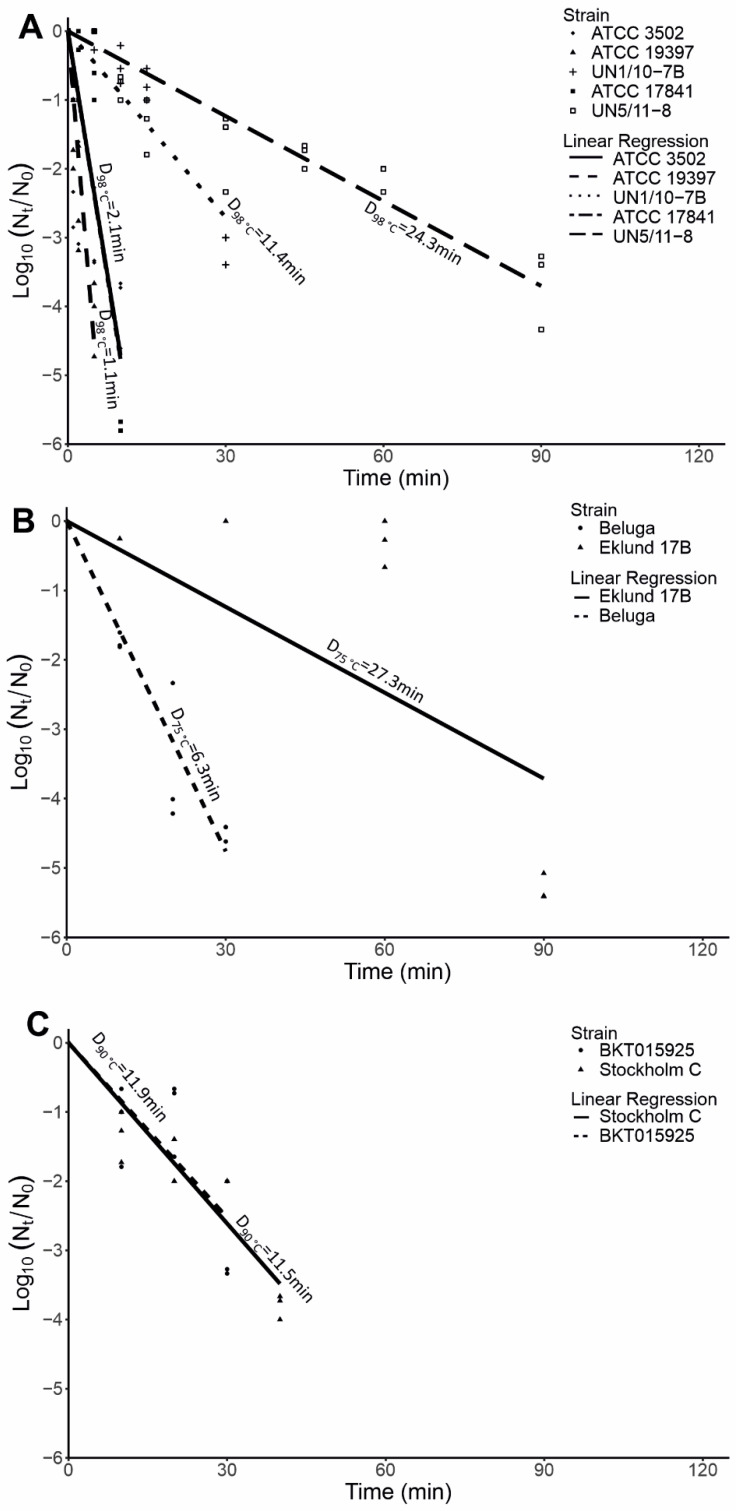
Thermal destruction of *Clostridium botulinum* spores. Purified spore suspensions of OD_600nm_~1 were subjected to (**A**) 98 °C for Group I strains, (**B**) 75 °C for Group II strains, (**C**) and 90 °C for Group III strains until a 3-log kill was achieved.

We tested the heat resistance of purified *C. botulinum* spores. As expected, Group I spores showed a relatively high heat resistance with D_98°C_ values varying from 1.1 min to 24.3 min, Group III spores showed a moderate heat resistance with D_90°C_ values of 11.5 min and 11.9 min, and Group II spores showed a relatively low resistance with D_75°C_ values of 6.3 min and 27.3 min. Within Group I, we observed an interesting pattern: isolates from recent botulism cases (UN1/10-7B and UN5/11-8) showed substantially higher D_98°C_ values (11.4–24.3 min) than the laboratory strains (ATCC 3502, ATCC 1784, and ATCC 19397, 1.1–2.1 min) (Figure 8A). Within Group II, we observed a four-fold difference between the D_75°C_ of Eklund 17B (27.3 min) and Beluga (6.3 min) (Figure 8B). Both Group III strains had similar D_90°C_ values (11.9 min and 11.5 min for BKT015925 and Stockholm C, respectively) (Figure 8C).

## 3. Discussion

Sporulation is a survival and pathogenesis strategy in bacteria. Producing a resistant cell type allows spore-forming pathogens to disperse and infect new hosts. The spores are dormant, which incurs a loss of individuals actively contributing to the population growth and pathogenesis. Therefore, each sporulating organism needs to adopt a sporulation strategy that ensures a balance between survival and growth in prevailing conditions. We studied the population-level sporulation strategies of *C. botulinum* strains by following their sporulation with phase contrast microscopy. Overall, the studied strains displayed two main strategies for sporulation. While the times when sporulating cells were first observed were similar for most strains, Group I strains showed an accumulation of cells in the late stages of sporulation, delaying the release of mature spores into cultures. By contrast, Group II and III cultures had small sporulating populations that released mature spores as soon as 48 h after inoculation. *C. botulinum* Groups I, II, and III are diverse species; thus, it is tempting to speculate that the different population-level sporulation strategies have evolved in adaptation to different metabolisms and natural environments. The proteolytic metabolism of Group I strains allows them to obtain more nutrients via proteolysis than non-proteolytic strains, which might afford them a longer sporulation time and a larger proportion of sporulating individuals. For the non-proteolytic, Group II and some Group III strains, it appears to be beneficial to promptly guarantee survival of a small part of the population.

We observed discontinuous spore coat formation in all strains studied. This is a common mechanism in clostridia; however, its timing during sporulation varies between species [29,43,44,45]. For example, *C. tetani* and *C. histolyticum* show spore coat fragments during engulfment [42]. In all our *C. botulinum* Group I, II, and III strains, we observed the coat fragments appearing after completed engulfment when a cortex was already visible. This suggests that, in *C. botulinum*, activation of coat gene expression is temporally regulated, as is the case in *B. subtilis* [46].

We identified four spore morphotypes through TEM. *C. botulinum* Groups I and III showed their own consistent morphotypes. Group I spores had thick and loose-fitting exosporia, while Group III strains displayed spores with thin and tight-fitting exosporia.

The three Group II strains showed three different morphotypes. Eklund 17B spores were similar to the spores of Group I strains, Beluga spores presented with antennae protruding from the spore coat and no exosporium, and CB11/1-1 spores showed both antennae and exosporium. This last morphotype has previously been reported as the spore morphology of Group II, type E strains, including Beluga, using spores obtained with lytic enzymes and not by natural mother cell lysis [30]. The apparently contradictory findings on the presence of an exosporium in Beluga can be explained by the loss or destruction of an exosporium during mother cell lysis. Alternatively, the structure observed in [30] was an artefact of enzymatic cell lysis. This assumption might be supported by the lack of *csxA* encoding CsxA, an essential protein for the assembly of the exosporium in *C. sporogenes* [47,48], in the Beluga genome, inferring that a proper exosporium is likely not formed. It is of note that *csxA* homologs can be found in the genomes of several *C. botulinum* Group I strains and Eklund 17B [48]. Interestingly, other exosporium-related genes identified in *C. sporogenes* were not found in the genomes of the Eklund 17B strain [48], suggesting that Group II exosporia composition differs from *C. sporogenes* and *C. botulinum* Group I strains.

Previous studies have shown moderate hydrophobicity of spores of *C. botulinum* Group I strain 213B [49]. All our purified *C. botulinum* Group I, II, and III spore suspensions displayed high or very high hydrophobicity, regardless of the spore morphotypes. Similarly, regarding autoaggregation, we did not observe differences between spore morphotypes. It is possible that spores with similar morphology have similar structural spore proteins, but adhesion proteins on their surface differ. Interestingly, all strains that displayed active autoaggregation were originally isolated from botulism cases, while strains with no or little autoaggregation were from environmental sources. While aggregation might assist in infectious processes, such as being toxicoinfectious or wound botulism, its possible relevance in the aetiology of foodborne botulism, which is an intoxication, remains unclear.

In line with previous knowledge, spores of each *C. botulinum* group displayed different levels of heat resistance, with Group I spores being the most heat resistant, Group II being the least heat resistant [22], and Group III showing moderate heat resistance [50]. The observed D-values, however, were mostly lower than those previously reported, which is likely due to the spore purification used. Spores for thermal destruction assays used for food safety testing were often prepared by using sonication and washes of the spore stock without further density gradient purification of spores from culture debris. Density gradient-purified spore suspensions lack the heat protection effects that cell debris and sugar, present in culture media, confer to the spores.

In both *C. botulinum* Group I and II spore suspensions, we observed two different patterns of heat resistance, with high and low D-values for strains within the same group. Spores of strains with the lower D-values within a group possessed no (Group II) or a very thin exosporium (Group I). Additionally, the strains with higher D-values within a group had larger spores (total spore size) than strains with lower D-values. Meanwhile, spores of both Group III strains had a similar size, exosporium thickness, and D-value. It appears that the exosporium contributes to heat resistance, probably by presenting an additional barrier to protect the spore core from heat, as well as by enlarging the spore size, which can potentially affect the heat distribution. However, further studies encompassing more strains and exosporium gene knockout mutants would bring insights into the role of the exosporium in the heat resistance of *C. botulinum* spores.

While the heat resistance of Groups I and II *C. botulinum* spores has been widely studied for food safety purposes, studies on the heat resistance of Group III spores unrelated to human disease are scarce. An early study demonstrated different heat resistance patterns for Group III spores of terrestrial and marine origin, with terrestrial spores showing higher heat resistance than marine spores [50]. Both of our Group III strains could be characterized as terrestrial and showed resistance patterns similar to [50]. To better understand the diverse spore heat resistance and its mechanisms in *C. botulinum* strains, a genome-wide association analysis of a large number of strains would be invaluable.

To conclude, we provide a systematic characterization of sporulation and spores of *C. botulinum* Groups I, II, and III strains. *C. botulinum* appears to have two distinct sporulation strategies, where the proteolytic, Group I strains take longer to sporulate and reach larger numbers of free spores than the non-proteolytic, Group II and III strains. The morphological stages of sporulation appeared to be similar in all strains. We observed discontinuous coat formation after engulfment and initiation of cortex formation, which suggests tight temporal regulation of coat gene expression. Four spore morphotypes were identified: one for Group I strains, one for Group III strains, and two for Group II strains. Each Group II strain had its own morphotype with one strain sharing the Group I morphotype. The differences in morphotypes did not appear to play a role in spore hydrophobicity or autoaggregation, with autoaggregation displayed only by strains isolated from botulism cases. Finally, the exosporium appeared to play a role in spore heat resistance as both the presence of an exosporium and its thickness seemed to confer heat resistance.

Our work expands on the knowledge of *C. botulinum* sporulation and spore biology and highlights the heterogeneity of spore heat resistance within each group, providing novel insights in the fields of food and feed safety and epidemiology.

## 4. Materials and Methods

### 4.1. Bacterial Strains and Growth Conditions

The present work included 10 *C. botulinum* strains belonging to Group I, Group II, and Group III (Table 2). The strain selection included historical and representative strains from each metabolic group, as well as more recent isolates from human botulism cases. We also selected strains producing different toxin type within each group.

All strains were cultured in anaerobic conditions (atmosphere of 85% N_2_, 10% CO_2_, and 5% H_2_) in an anaerobic workstation (MACS MG-1000, Don Whitley, Bingley, UK). Group I strains were propagated in tryptone-peptone-glucose-yeast extract (TPGY) broth at 37 °C. Group II strains were grown in TPGY broth and a bi-phasic sporulation medium (Cooked Meat Medium Agar + TPGY) at 30 °C [26]. Finally, Group III strains were grown at 37 °C in TPGY supplemented with 0.2% (*w*/*v*) L-cysteine [51].

### 4.2. Phase Contrast Microscopy

Sporulation of *C. botulinum* cultures was monitored by phase contrast microscopy. One milliliter of each culture was pelleted, washed, and re-suspended in 100 µL of anaerobic phosphate buffered saline buffer (PBS, Sigma-Aldrich, St. Louis, MO, USA). Three microliters of washed bacterial preparation was spotted onto an agarose slide and mounted samples were observed using a Leica DMi8 microscope (Leica Microsystems GmbH, Wetzlar, Germany) equipped with a 100X objective and an Orca-Flash4.0LT camera (Hamamatsu Photonics K. K., Iwata City, Japan). Acquired images were further processed and analyzed with MetaMorph (version 7.10.0.119, Molecular Devices, San Jose, CA, USA). Cells were classified and counted according to their morphology as follows: non-sporulating vegetative cells, sporulating cells, free phase-bright spores, and free phase-dark spores. Bacterial cells that were out of focus, overlapped, or not entirely located within the boundaries of the image field were excluded from the analysis.

### 4.3. Spore Purification

For each strain, 50 mL of the appropriate sporulation medium was inoculated from an overnight culture to an initial optical density at 600 nm (OD_600nm_) of ~0.05 (AU), and the OD_600nm_ was recorded over time (Figure S5). This was performed in triplicate (biological replicates).

At the mid-logarithmic growth phase (5 h for Group I strains and 8 h for Group II and III strains) and at 24 h, 48 h, and 72 h post inoculation, the cultures were observed by phase contrast microscopy and sampled for further transmission electron microscopy analyses.

The total duration of sporulation was estimated to be 3 days for Group II and III strains and 10 days for Group I strains, according to phase contrast microscopy observations (Figure S6). Once the sporulation was completed, free spores were harvested by centrifugation (10,000× *g*, 5 min, 4 °C) and washed twice with cold 0.1% (*v*/*v*) Triton X-100 (ICN Biomedicals Inc., Irvine, CA, USA). Washed spore pellets were then resuspended in 20% (*v/v*) solution of Gastrografin^®^ (Bayer AG, Leverkusen, Germany) and layered over 50% (*v*/*v*) Gastrografin^®^ solution in round-bottom tubes to create a density gradient. The gradient tubes were then centrifuged (10,000× *g*, 30 min, 4 °C) and the layer of debris and extra Gastrografin solution were removed from the tubes. The pellets were resuspended in cold 0.1% (*v*/*v*) Triton X-100 and transferred to a clean Eppendorf tube. Finally, the spore suspensions were washed five times with cold 0.1% (*v*/*v*) Triton X-100 (5000× *g*, 2 min, room temperature), and purity was assessed by phase contrast microscopy until >90% of the cells were free phase-bright spores and the amount of cell debris was minimal/absent. If needed, the gradient centrifugation and subsequent washes were repeated, as described above.

The purification process was affected by high numbers of free phase-dark spores, indicating re-germination or damage of newly formed spores. Three strains had a particularly high number of free phase-dark spores already at 72 h (Beluga 17.2 ± 7.4%, CB11/1-1 14.9 ± 7.8%, BKT015925 7.5 ± 6.2%; Table 1). This led us to set 72 h as the endpoint of the sporulation experiments for Group II and III cultures.

### 4.4. Transmission Electron Microscopy (TEM)

The ultrastructures of sporulating cells and spores were analyzed using TEM [20,59]. The samples were stained, dehydrated, embedded in either Epon resin (sporulating cells) or LV/PO resin (spores), cut, and mounted on copper grids (Electron Microscopy Unit, University of Helsinki). Sporulating cells were stained with 1% (*w*/*v*) osmiumtetroxide solution for 1 h, and spores were successively stained for 1 h with 0.01% (*w*/*v*) tannic acid solution, 1% (*w*/*v*) osmiumtetroxide solution, and 4% (*w*/*v*) uranylacetate solution with 5 min washes in sterile water in between. The grids were visualized using a Jeol 1400 microscope (Jeol Ltd., Tokyo, Japan). DigitalMicrograph (version 3.40.2804.0, Gatan Inc., Pleasanton, CA, USA) software was used for image acquisition and analysis.

### 4.5. Spore Measurements in Thin Section Samples

For each strain, we selected ten individual spores cut in their center following their longest axis. Their ultrastructures were visualized and measured using Adobe Photoshop CS6 version 13.0 (Adobe Systems Inc., Mountain View, CA, USA). Thirteen different measurements (Table S1) were collected for each spore. Length and width were measured at a single site per spore, while thickness was measured at three different sites per structural component per spore and averaged. The length of every single appendage present in each *C. botulinum* Group II Beluga spore was measured (Figure S4). One-way ANOVA (Post-Hoc test: Tukey HSD) was performed using IBM SPSS Statistics version 25 (IBM, Armonk, NY, USA) on the spore measurements, using the single measurements and averages thickness which were measured three times. All original measurements used for the analysis are presented in Table S2.

### 4.6. Spore Autoaggregation

The capacity of purified spores to autoaggregate and sediment was tested for each strain in triplicate [32]. Purified spore suspensions were adjusted to OD_600nm_~1. Initial OD_600nm_ was measured, and the suspensions were allowed to settle in OD cuvettes for 24 h without agitation. Estimations of the sedimentation velocity were achieved by acquiring still images of the spore suspensions every half hour with a Canon EOS 40D digital camera (Canon Inc., Tokyo, Japan). After 24 h, the upper fraction (top 80%) of the suspension was sampled, and the OD_600nm_ was measured. The percentage of sedimentation was calculated as Sedimentation (%) = 100 × [1 − (OD_f_/OD_i_)], where OD_f_ is the final OD_600nm_ and OD_i_ is the initial OD_600nm_. Additionally, in one of the replicate experiments, phase contrast images were acquired to monitor the changes in the number and dispersion of cells before and after allowing the suspensions to settle.

### 4.7. Spore Hydrophobicity

Hydrophobicity of the purified spores was tested by measuring the bacterial adherence to hydrocarbons (BATH) using a modified protocol [33]. Pure spore suspensions in sterile ddH_2_O were adjusted to OD_440nm_~0.5, and 100 µL of hexadecane (Sigma-Aldrich) was added to the suspension. Each cuvette was vortexed at maximum speed for 1 min and allowed to settle for 10 min to separate the aqueous and hexadecane phases. Using thin pipette tips, the aqueous phase was transferred to a clean cuvette, allowed to rest for an additional 10 min, and OD_440nm_ was measured. The percentage of spores captured by hexadecane was calculated as Hydrophobicity (%) = [100 × (OD_i_ − OD_f_)]/OD_i_, where OD_f_ is the final OD_440nm_ and OD_i_ is the initial OD_440nm_.

### 4.8. Spore Heat Resistance

Purified spore suspensions were adjusted to OD_600nm_~1 in sterile ddH_2_O, which, when using purified spore suspensions, translates to an approximate 10^8^ spore titer. To determine the initial spore titer, 150 µL of the spore suspensions was transferred into a sterile tube. The remaining suspension was aliquoted in replicate tubes to a volume of 150 µL. Samples were heat-treated in a water bath at 80 °C, 90 °C, or 98 °C for a duration ranging from 1 min to 120 min. After heat treatment, the tubes were cooled to room temperature and transferred to an anaerobic chamber. Most probable number (MPN) assays were performed as previously described to determine the initial and heat-treated spore titers [20]. Results were read after two days of incubation and D-values were calculated. Several time-temperature combinations were tested until we achieved at least a 3-log kill, a time/temperature combination where at least 10^3^ spores were inactivated. D-values were calculated by plotting the log_10_ (N_t_/N_0_) values from each individual replicate against time (min), where N_t_ and N_0_ were the MPN values after and before heating, respectively. Regression lines were fitted to each set of data, and the D-values were calculated using D_T_ = −1/*a*, where *a* is the slope of the regression line.

## Figures and Tables

**Table 1:** Enumeration of cell types of 10 *Clostridium botulinum* strains by phase contrast microscopy. The table shows the average and standard deviation of the percentage of cells belonging to three replicates except for UN1/10-7B strain (only two replicates).

Group	Strain	Hours	Non-Sporulating Cells	Sporulating Cells	Free Spores	Free Phase-Dark Spores	Total Cell Number
I	ATCC 3502	5	100 ± 0.0	0.0 ± 0.0	0.0 ± 0.0	0.0 ± 0.0	n = 1009
		24	96 ± 2.0	4.0 ± 2.0	0.0 ± 0.0	0.0 ± 0.0	n = 1108
		48	84 ± 7.1	16 ± 7.1	0.0 ± 0.0	0.0 ± 0.0	n = 2484
		72	79 ± 5.7	19 ± 5.5	1.4 ± 0.6	0.0 ± 0.0	n = 2829
	ATCC 19397	5	100 ± 0.0	0.0 ± 0.0	0.0 ± 0.0	0.0 ± 0.0	n = 798
		24	98 ± 0.3	1.2 ± 0.3	0.1 ± 0.2	0.0 ± 0.0	n = 1127
		48	95 ± 1.4	4.0 ± 1.2	0.4 ± 0.2	0.0 ± 0.0	n = 1044
		72	90 ± 0.7	6.6 ± 1.2	2.9 ± 0.8	0.0 ± 0.0	n = 1975
	UN1/10-7B	5	100	0.0	0.0	0.0	n = 692
		24	98	1.8	0.0	0.0	n = 853
		48	64	35	0.4	0.0	n = 716
		72	50	48	0.2	0.0	n = 757
	ATCC 17841	5	100 ± 0.2	0.0 ± 0.0	0.2 ± 0.2	0.0 ± 0.0	n = 1636
		24	98 ± 1.6	2.1 ± 1.5	0.1 ± 0.1	0.0 ± 0.0	n = 1281
		48	75 ± 11	25 ± 12	0.2 ± 0.2	0.0 ± 0.0	n = 1721
		72	54 ± 21	42 ± 21	2.9 ± 1.7	0.3 ± 0.2	n = 1116
	UN5/11-8	5	100 ± 0.1	0.0 ± 0.1	0.0 ± 0.0	0.0 ± 0.0	n = 1553
		24	99 ± 0.4	1.0 ± 0.4	0.0 ± 0.0	0.0 ± 0.0	n = 3510
		48	88 ± 2.4	11 ± 2.2	1.3 ± 0.3	0.0 ± 0.0	n = 1083
		72	90 ± 1.3	6.8 ± 1.6	3.4 ± 0.4	0.0 ± 0.0	n = 1219
II	Eklund 17B	8	Low cell density, no pellet was obtained				
		24	100 ± 0.2	0.1 ± 0.2	0.0 ± 0.0	0.0 ± 0.0	n = 2166
		48	75 ± 4.2	6.8 ± 1.8	18 ± 5.9	0.1 ± 0.2	n = 1036
		72	76 ± 4.3	0.3 ± 0.1	22 ± 4.1	0.9 ± 0.8	n = 1208
	Beluga	8	98 ± 1.3	2.2 ± 1.3	0.0 ± 0.0	0.0 ± 0.0	n = 3390
		24	80 ± 3.8	4.8 ± 1.4	15 ± 4.2	0.0 ± 0.0	n = 831
		48	38 ± 1.1	5.6 ± 2.2	56 ± 1.9	0.0 ± 0.0	n = 370
		72	7.7 ± 4.5	0.5 ± 0.9	75 ± 4.6	17 ± 7.4	n = 550
	CB11/1-1	8	Low cell density, no pellet was obtained				
		24	100 ± 0.0	0.0 ± 0.0	0.0 ± 0.0	0.0 ± 0.0	n = 1346
		48	85 ± 1.0	3.8 ± 1.4	9.0 ± 1.5	2.5 ± 0.6	n = 1138
		72	60 ± 18	1.9 ± 0.9	23 ± 12	15 ± 7.8	n = 1130
III	Stockholm C	8	100 ± 0.0	0.0 ± 0.0	0.0 ± 0.0	0.0 ± 0.0	n= §
		24	94 ± 0.5	3.1 ± 1.4	3.0 ± 1.4	0.0 ± 0.0	n = 829
		48	82 ± 2.6	7.4 ± 1.5	8.9 ± 1.2	1.5 ± 1.0	n = 788
		72	60 ± 2.2	11 ± 4.4	27 ± 3.1	1.2 ± 1.1	n = 457
	BKT015925	8	100 ± 0.1	0.2 ± 0.1	0.0 ± 0.0	0.0 ± 0.0	n = 2603
		24	94 ± 1.3	1.2 ± 0.8	4.9 ± 1.2	0.0 ± 0.0	n = 899
		48	65 ± 21	1.7 ± 0.7	33 ± 21	0.0 ± 0.0	n = 302
		72	46 ± 5.0	1.1 ± 2.0	45 ± 6.4	7.5 ± 6.2	n = 427

§ In early time points, Stockholm C formed extremely long cells that divided into normal-sized cells. It proved to be difficult to determine which cells were individual cells and which cells were still part of an elongated cell. However, no cells showed signs of sporulation.

**Table 2:** *C. botulinum* strains used in this work.

Group	Toxin Type	Name	Source	Proteolytic	Growth/Sporulation Media
I	A	ATCC 3502	Historical strain, likely foodborne botulism, early 1920s (USA) [52]	Yes	TPGY
	A	ATCC 19397	Historical strain, likely foodborne botulism, before 1947 (USA) [52]	Yes	TPGY
	A	UN1/10-7B	Infant botulism, 2010 (Finland) [53]	Yes	TPGY
	B	ATCC 17841	Unknown origin, ATCC collection	Yes	TPGY
	B	UN5/11-8	Almond-filled, olive causing foodborne botulism, 2011 (Finland) [54]	Not known	TPGY
II	B	Eklund 17B	Marine sediment, 1965 [55]	No	TPGY/CMM-TPGY
	E	Beluga	Fermented whale flippers, 1951 (Alaska) [56]	No	TPGY/CMM-TPGY
	E	CB11/1-1	Whitefish eggs causing foodborne botulism, 1999 (Finland) [57]	No	TPGY/CMM-TPGY
III	C	Stockholm C	Mink farm, 1949 (Sweden) [58]	Not known	TPGY-Cys
	CD	BKT015925	Chicken liver, 2008 (Sweden) [51]	Not known	TPGY-Cys

## Data Availability

Not applicable.

## Supplementary Materials

The following are available online at https://www.mdpi.com/article/10.3390/ijms23020754/s1.
