# Supplementary material for: Sporulation Strategies and Potential Role of the Exosporium in Survival and Persistence of Clostridium botulinum
Source: Int J Mol Sci. 2022 Jan 11;23(2):754. doi: 10.3390/ijms23020754 (PMC8775613; doi:10.3390/ijms23020754)
Supplement: Supplementary file 1 [file ijms-23-00754-s001.zip › ijms-1489192-supplementary.pdf]

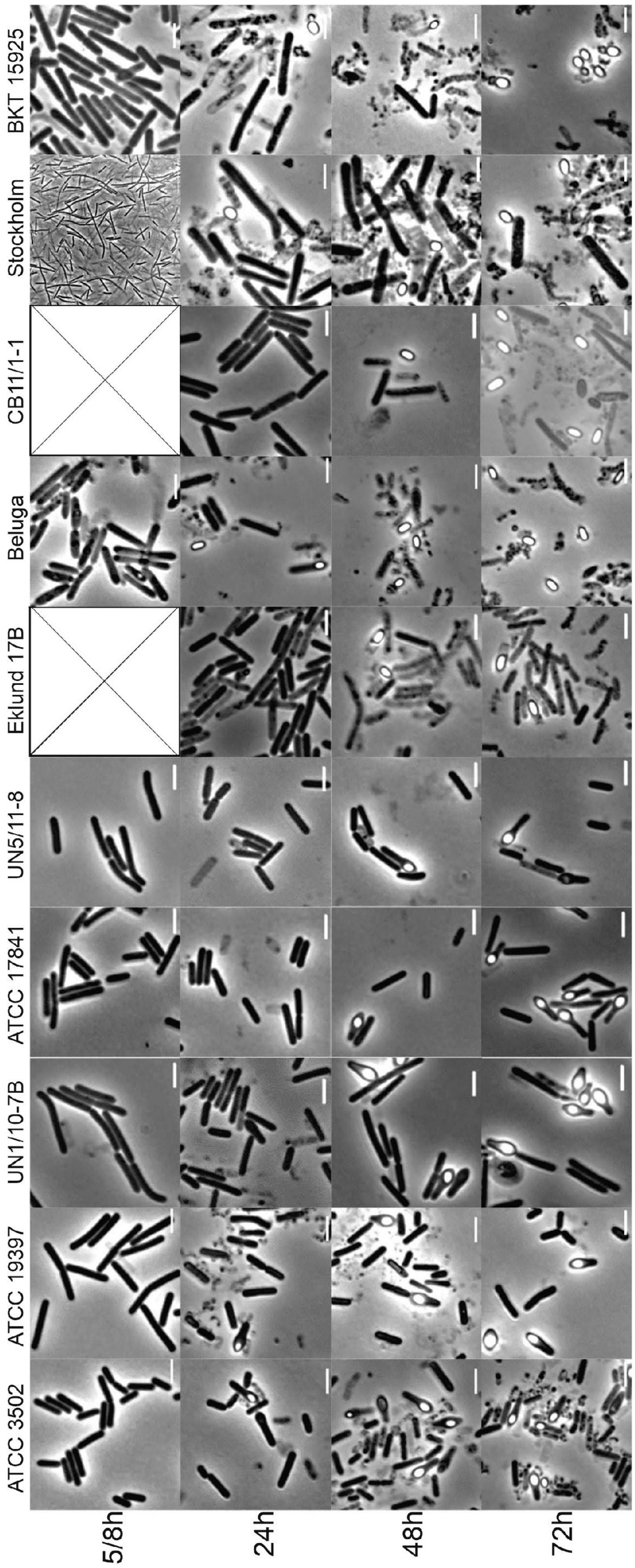

Figure S1. Phase contrast microscopy of sporulating *Clostridium botulinum* cultures. Images were acquired at the same time as samples were taken for TEM images. The earliest timepoint was five hours for Group I strains and eight hours for Group II and III strains. CB11/1-1 and Eklund 17B do not have images for the earliest time point as the culture density was too low to obtain a pellet for microscopy. Scale bars represent 1 μm.

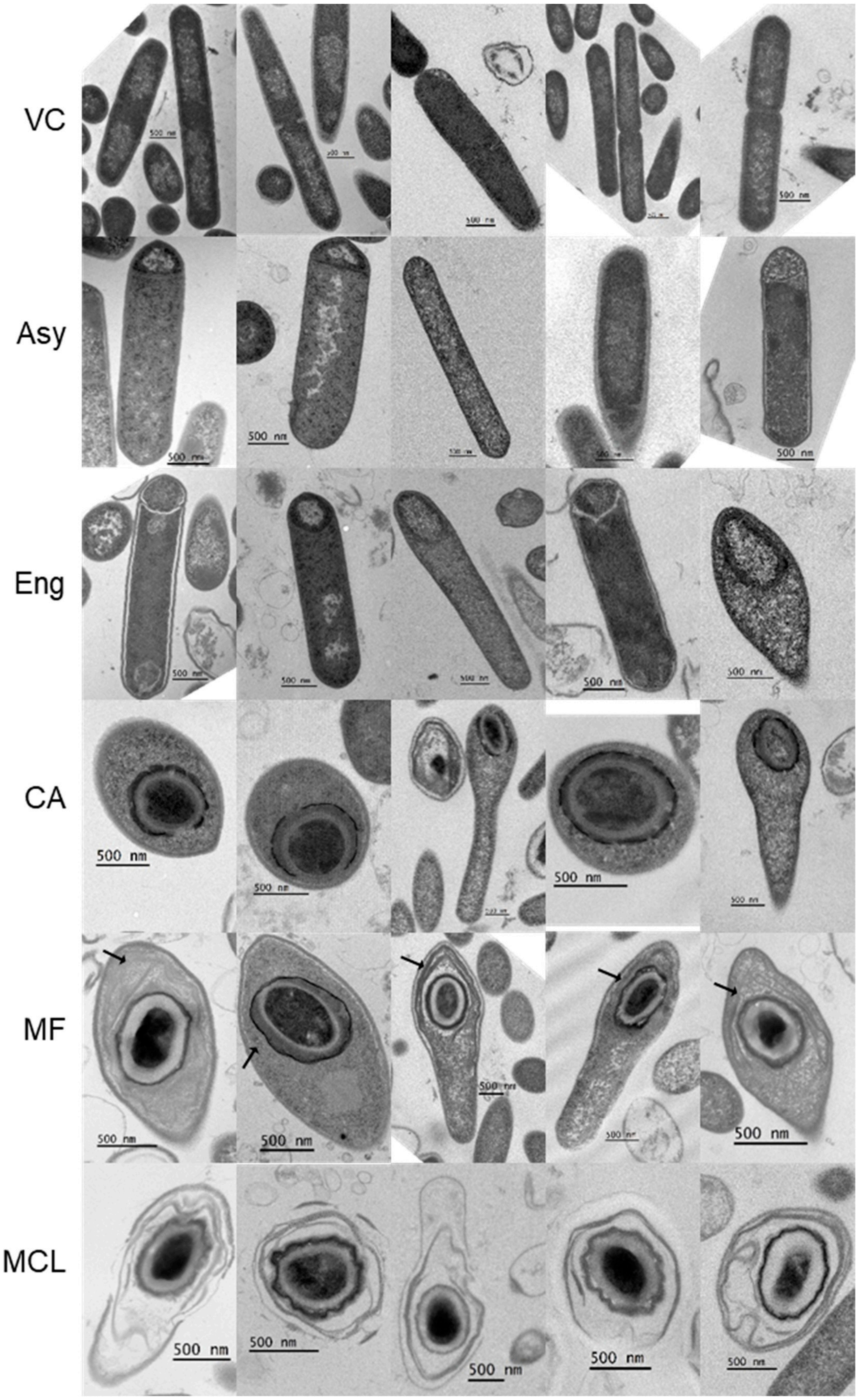

Figure S2. Enlarged electron micrographs of sporulating cells of *Clostridium botulinum* Group I strains. The cells were categorized according to their morphological stages: VC – Vegetative cell, Asy – Asymmetric division, Eng – Engulfment, CA – Coat assembly, MF – Mature forespore, MCL – mother cell lysis. Exosporia visible, while the forespore is still in the mother cell, are indicated with black arrows. Scale bars represent 500 nm.

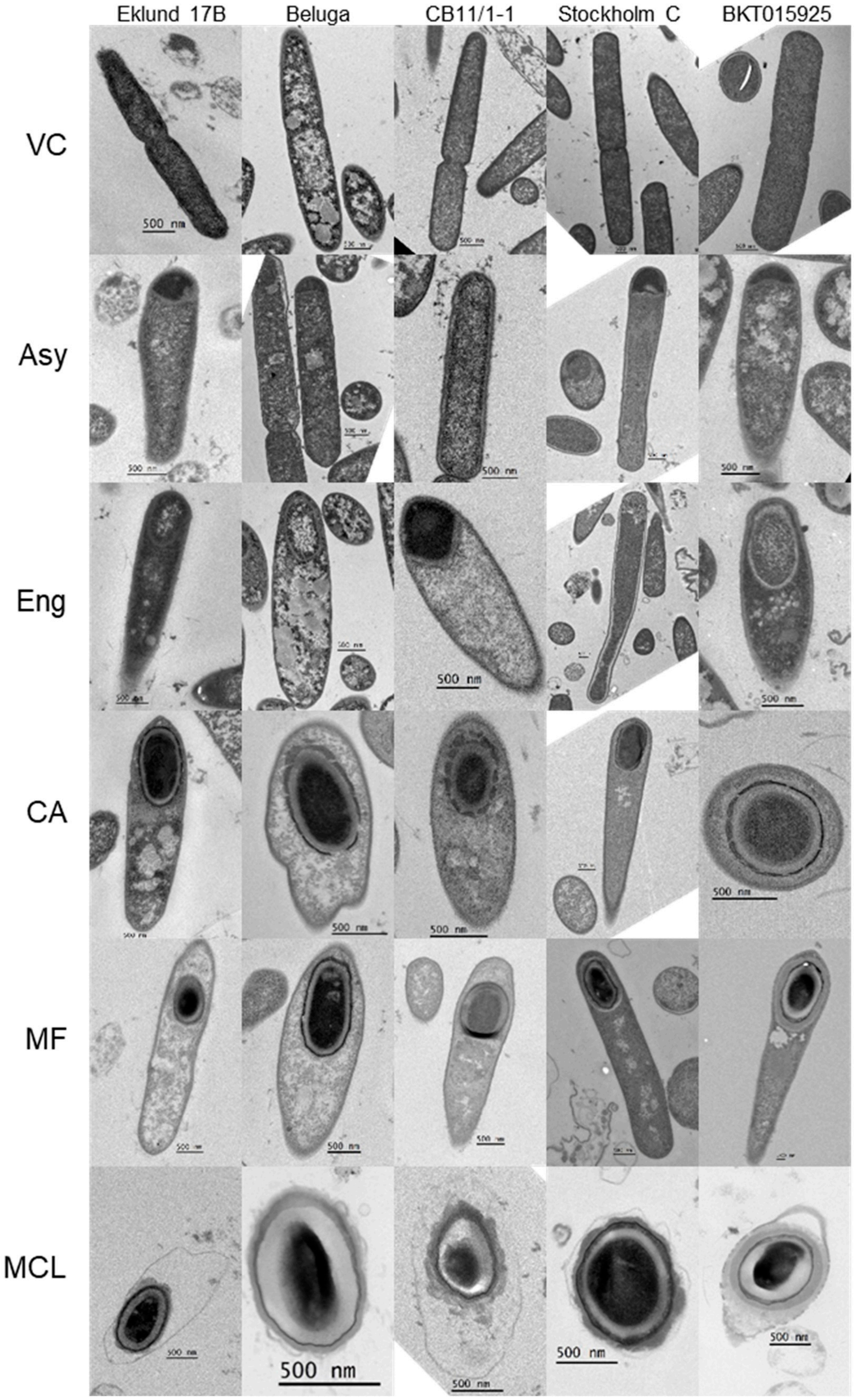

Figure S3. Enlarged electron micrographs of sporulating cells of *Clostridium botulinum* Group II and III strains. The cells were categorized according to their morphological stages: VC – Vegetative cell, Asy – Asymmetric division, Eng – Engulfment, CA – Coat assembly, MF – Mature forespore, MCL – mother cell lysis. Exosporia visible, while the forespore is still in the mother cell, are indicated with black arrows. Scale bars represent 500 nm.

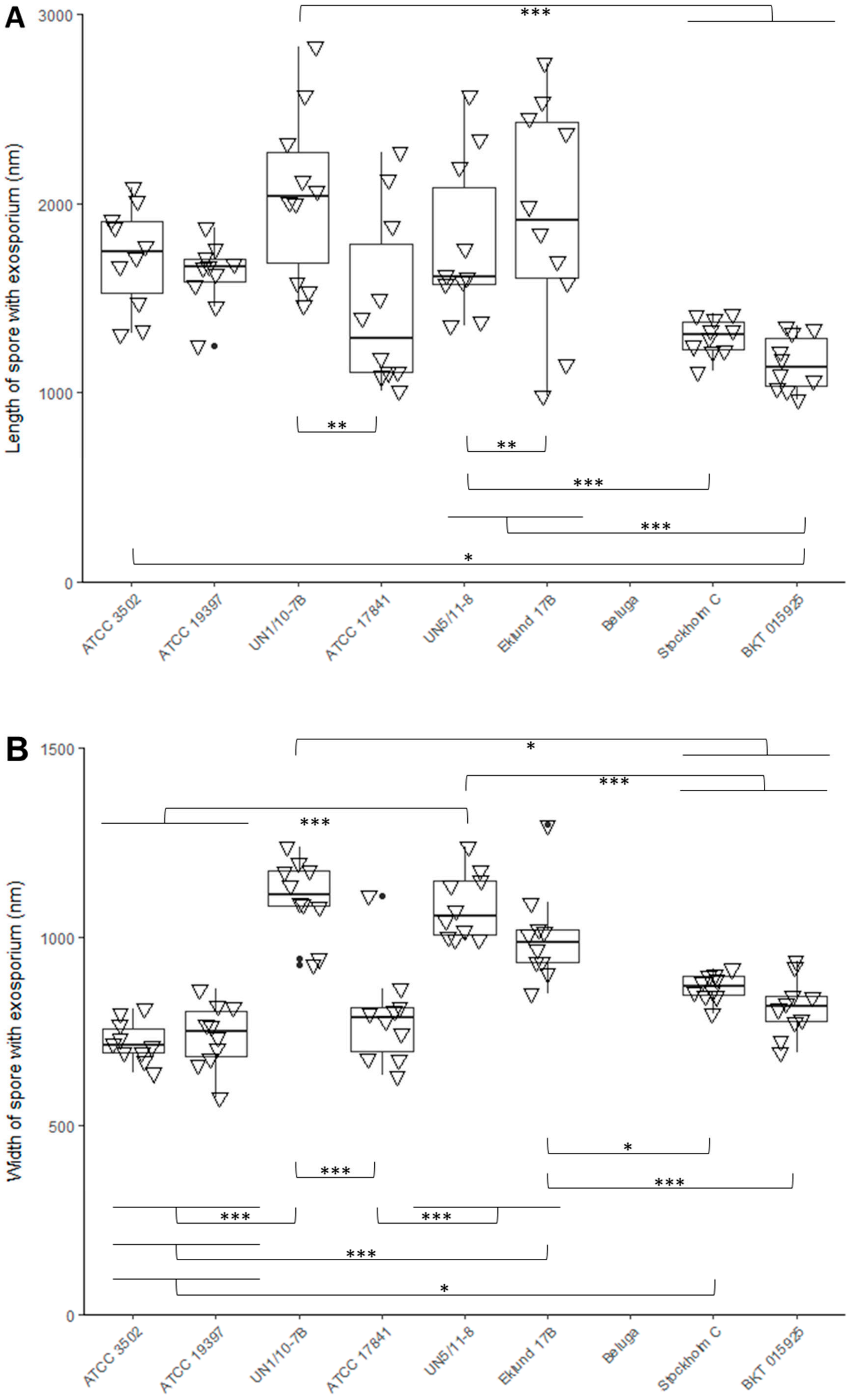

A
B
Length of spore with exosporium (nm)
Width of spore with exosporium (nm)
0
1000
2000
3000
500
1500
***
**
*
ATCC 3502
ATCC 19397
UN1/10-7B
ATCC 17841
UN5/11-8
Eklund 17B
Beluga
Stockholm C
BKT 015925

C

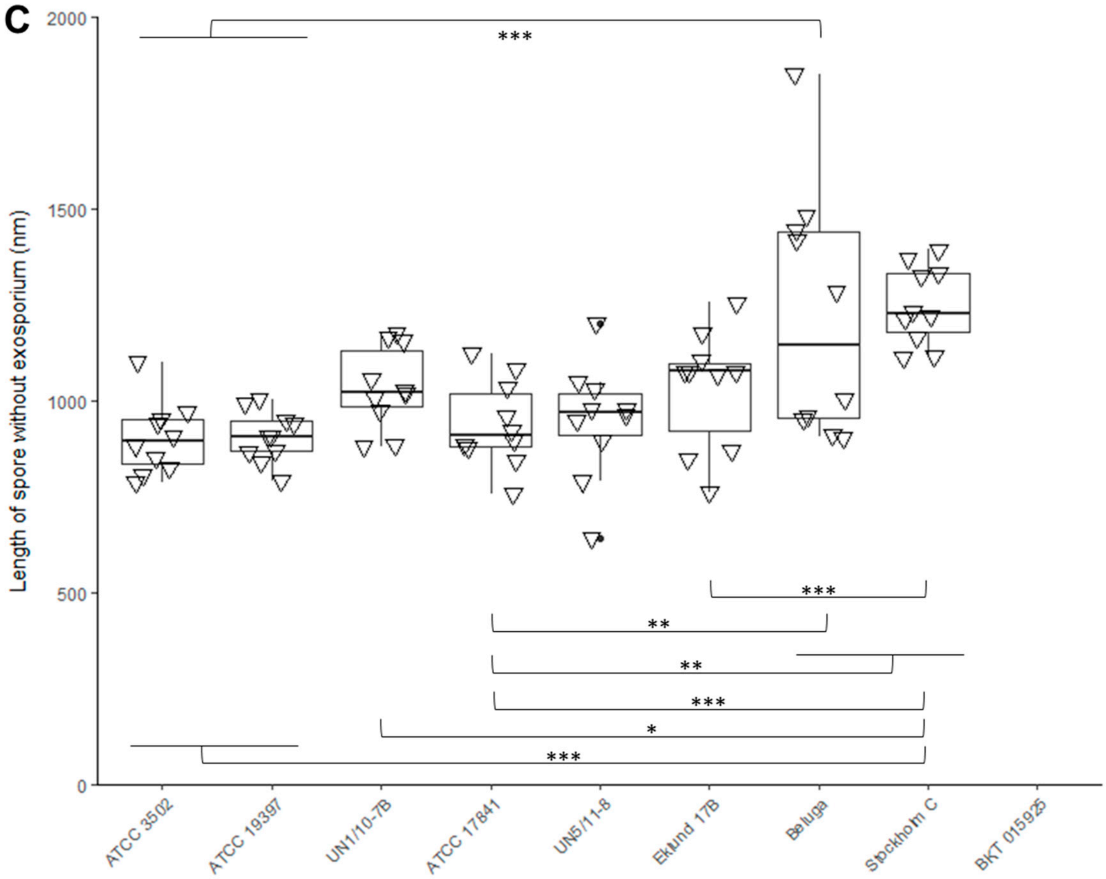

2000
1500
1000
500
0
Length of spore without exosporium (nm)
***
***
**
**
***
*
***
ATCC 3502
ATCC 19397
UN1/10-7B
ATCC 17841
UN5/11-8
Eklund 17B
Beluga
Stockholm C
BKT 015925

D

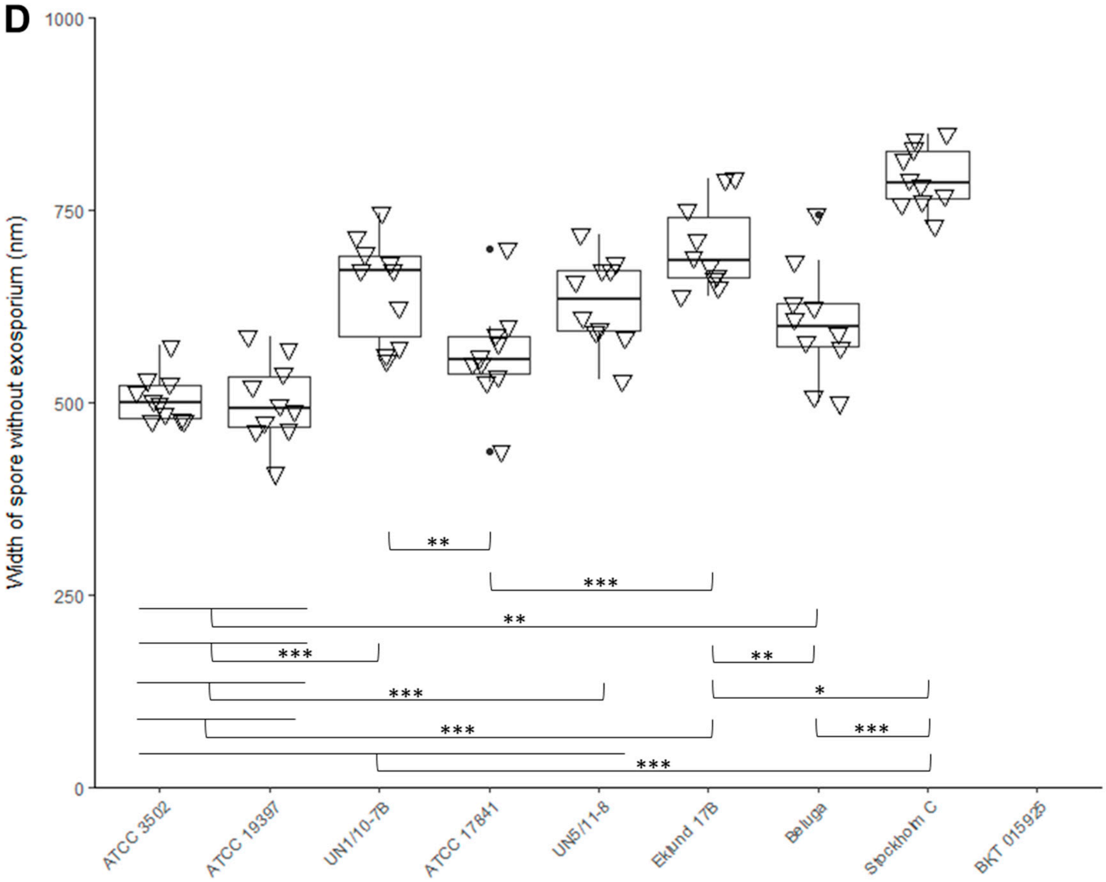

1000
750
500
250
0
Width of spore without exosporium (nm)
**
***
**
***
**
***
*
***
***
***
ATCC 3502
ATCC 19397
UN1/10-7B
ATCC 17841
UN5/11-8
Eklund 17B
Beluga
Stockholm C
BKT 015925

E

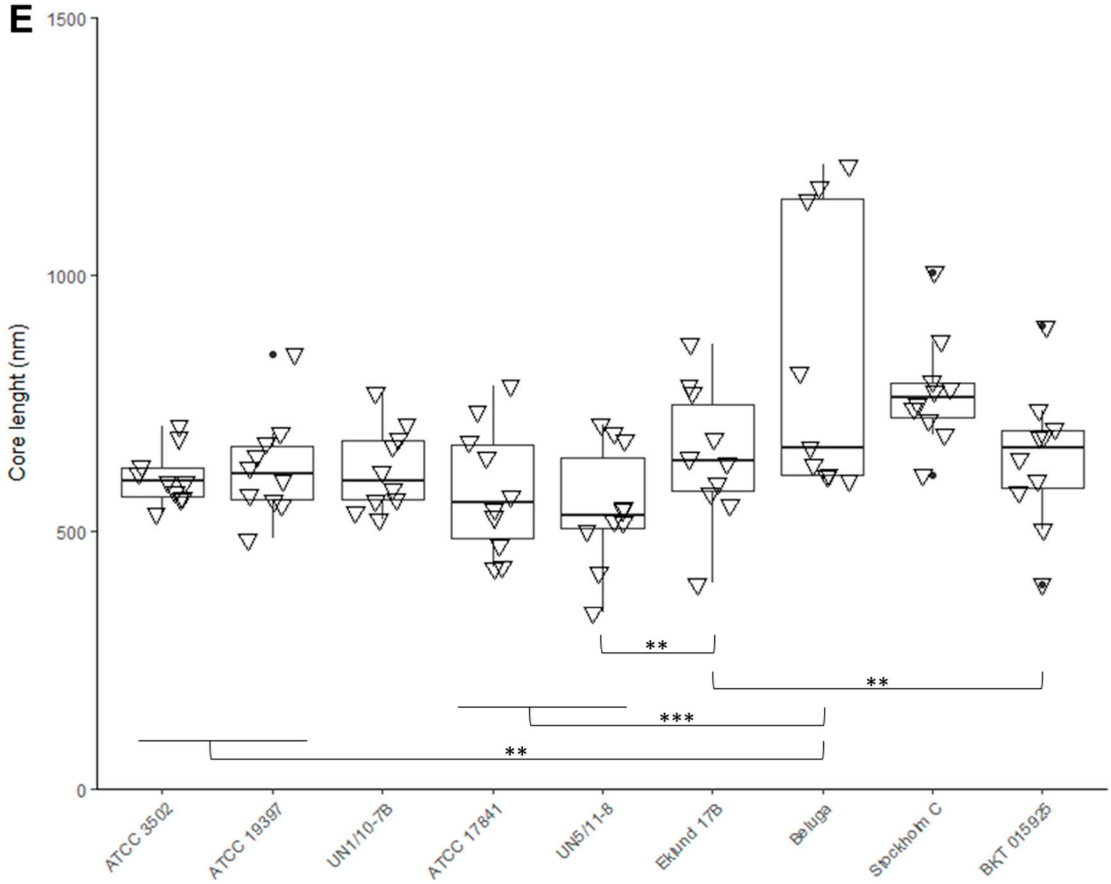

1500
1000
500
0
Core lenght (nm)
**
**
***
**
ATCC 3502
ATCC 19397
UN1/10-7B
ATCC 17841
UN5/11-8
Eklund 17B
Beluga
Stockholm C
BKT 015925

F

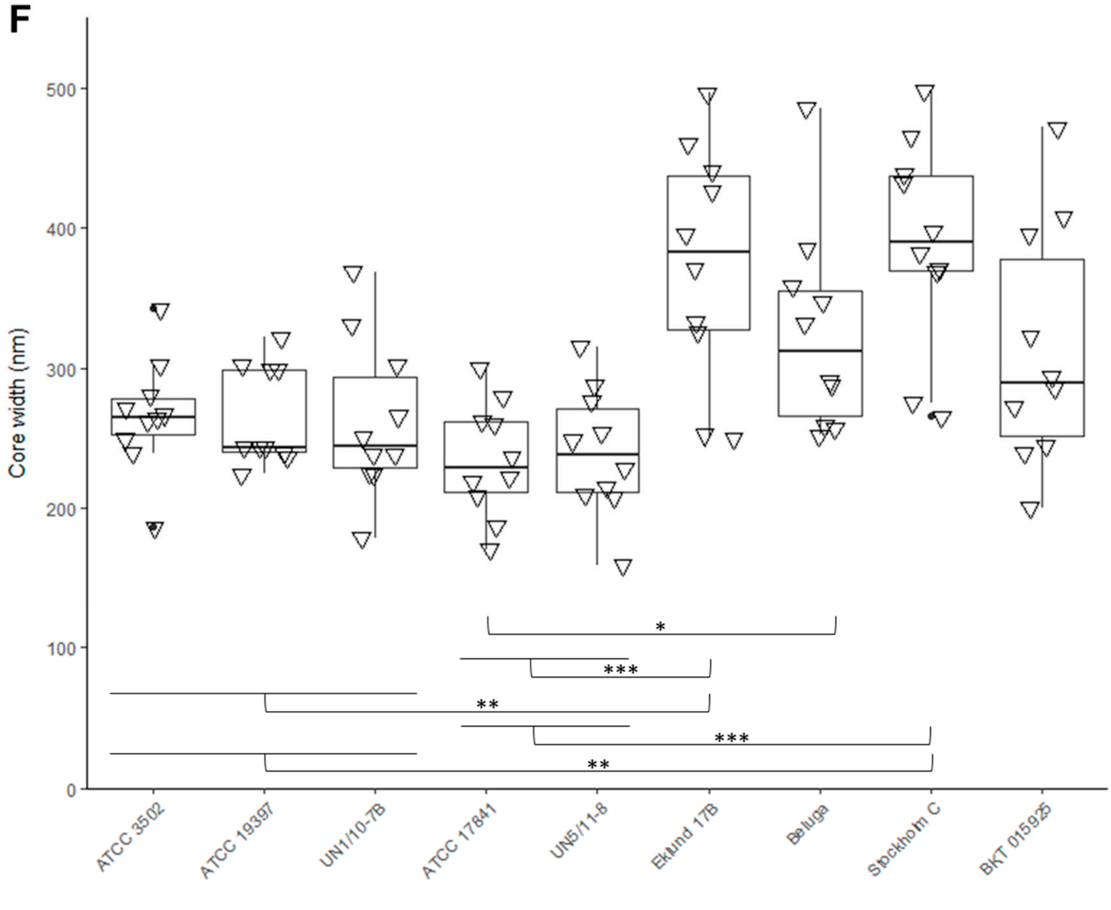

500
400
300
200
100
0
Core width (nm)
*
***
**
***
**
ATCC 3502
ATCC 19397
UN1/10-7B
ATCC 17841
UN5/11-8
Eklund 17B
Beluga
Stockholm C
BKT 015925

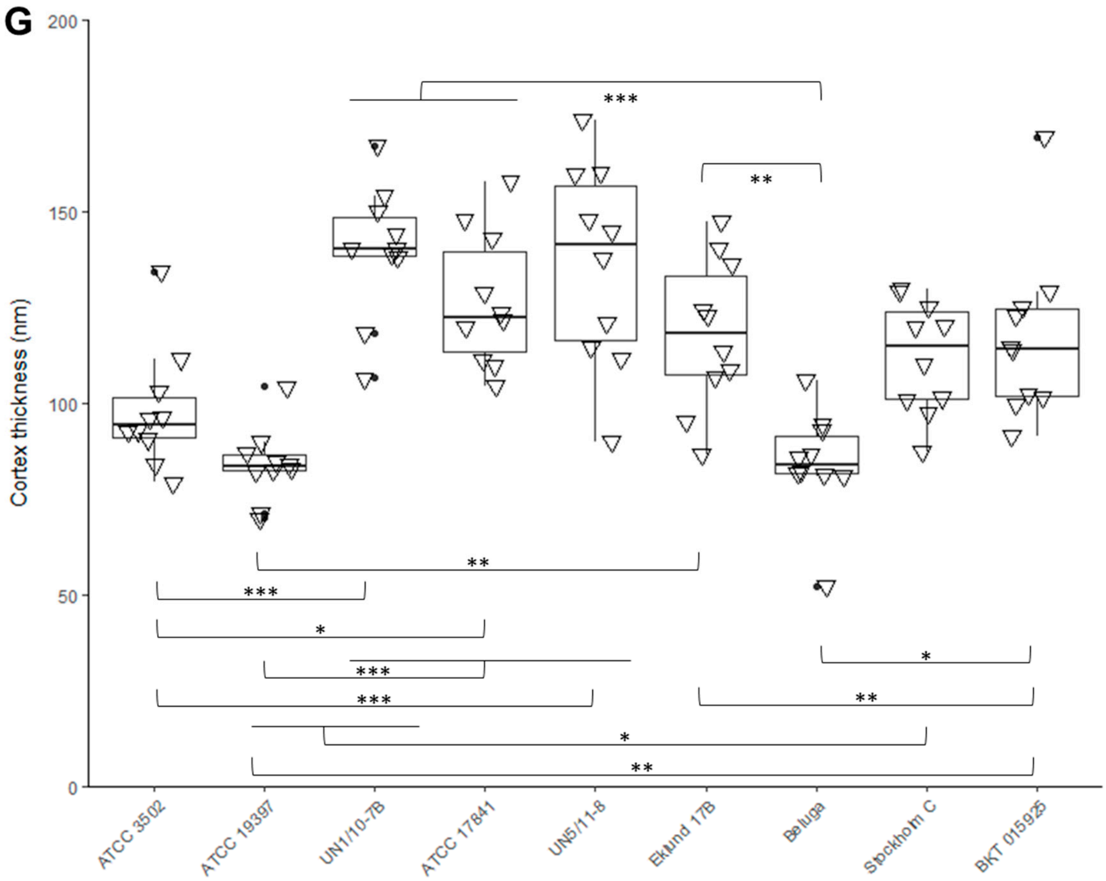
G
Cortex thickness (nm)
200
150
100
50
0
***
**
***
*
***
***
*
**
*
**
ATCC 3502
ATCC 19397
UN1/10-7B
ATCC 17841
UN5/11-8
Eklund 17B
Beluga
Stockholm C
BKT 015925

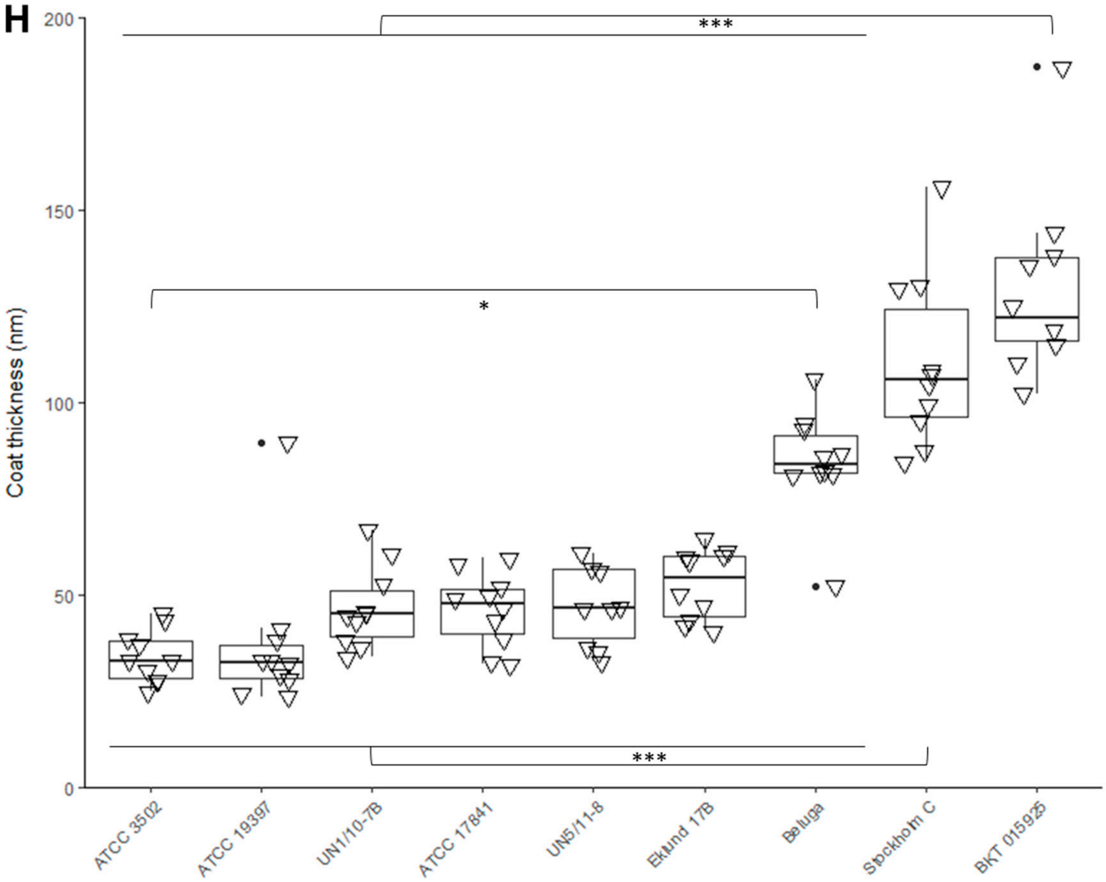
H
Coat thickness (nm)
200
150
100
50
0
***
*
***
ATCC 3502
ATCC 19397
UN1/10-7B
ATCC 17841
UN5/11-8
Eklund 17B
Beluga
Stockholm C
BKT 015925

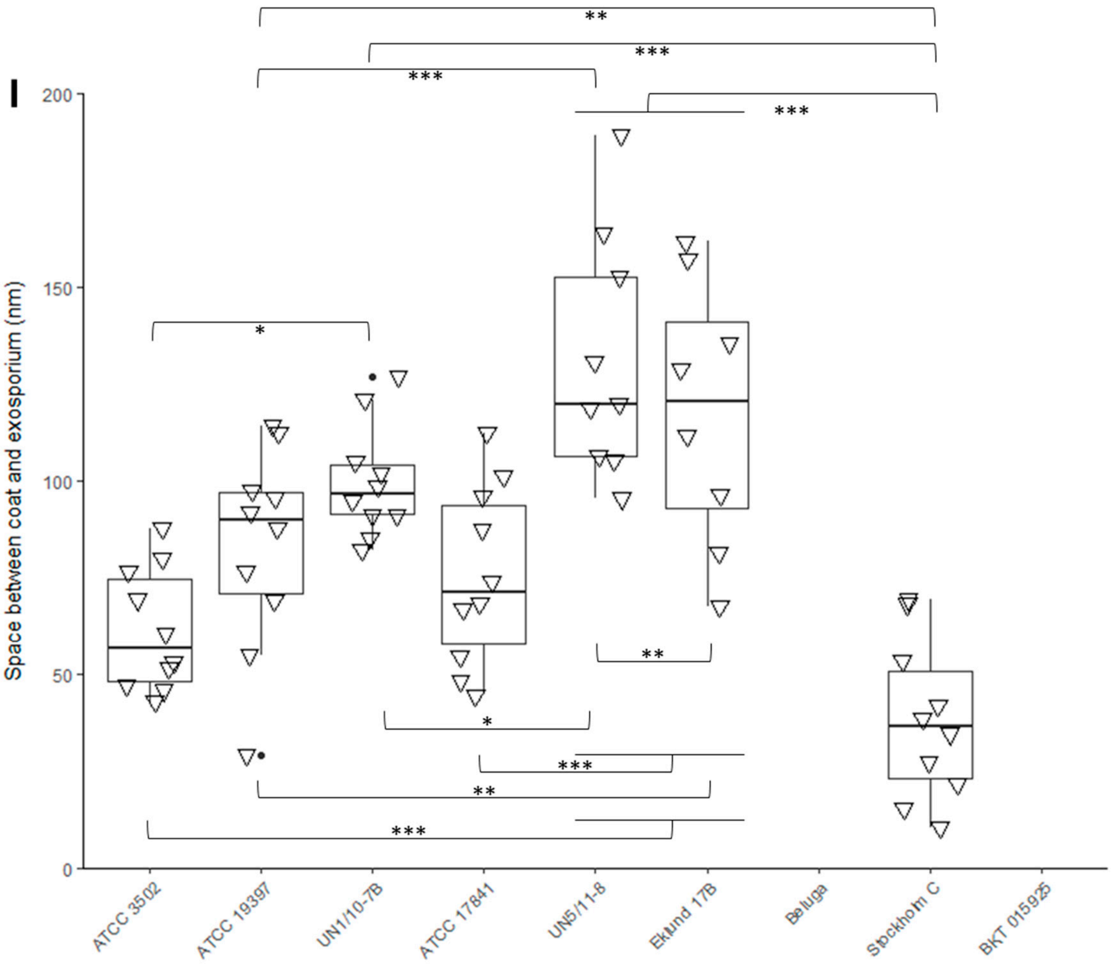
I
Space between coat and exosporium (nm)
ATCC 3502
ATCC 19397
UN1/10-7B
ATCC 17841
UN5/11-8
Eklund 17B
Beluga
Stockholm C
BKT 015925

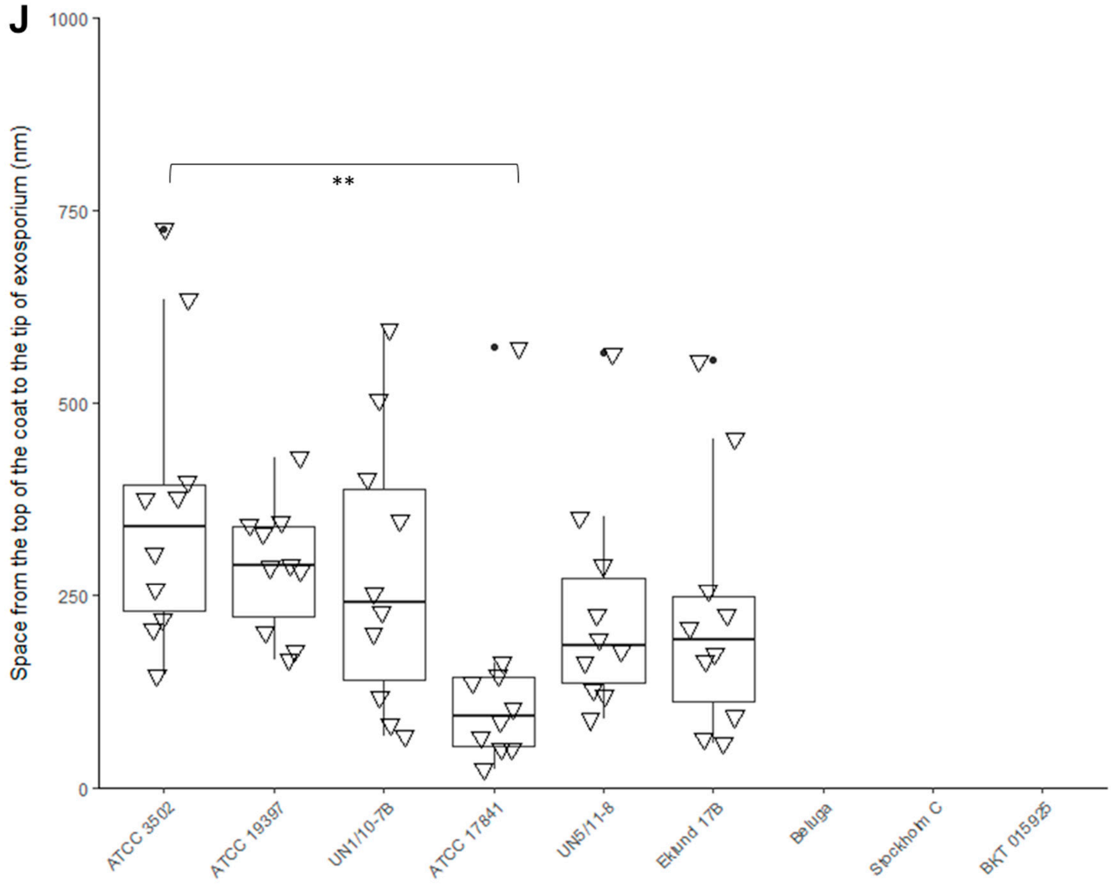
J
Space from the top of the coat to the tip of exosporium (nm)
ATCC 3502
ATCC 19397
UN1/10-7B
ATCC 17841
UN5/11-8
Eklund 17B
Beluga
Stockholm C
BKT 015925

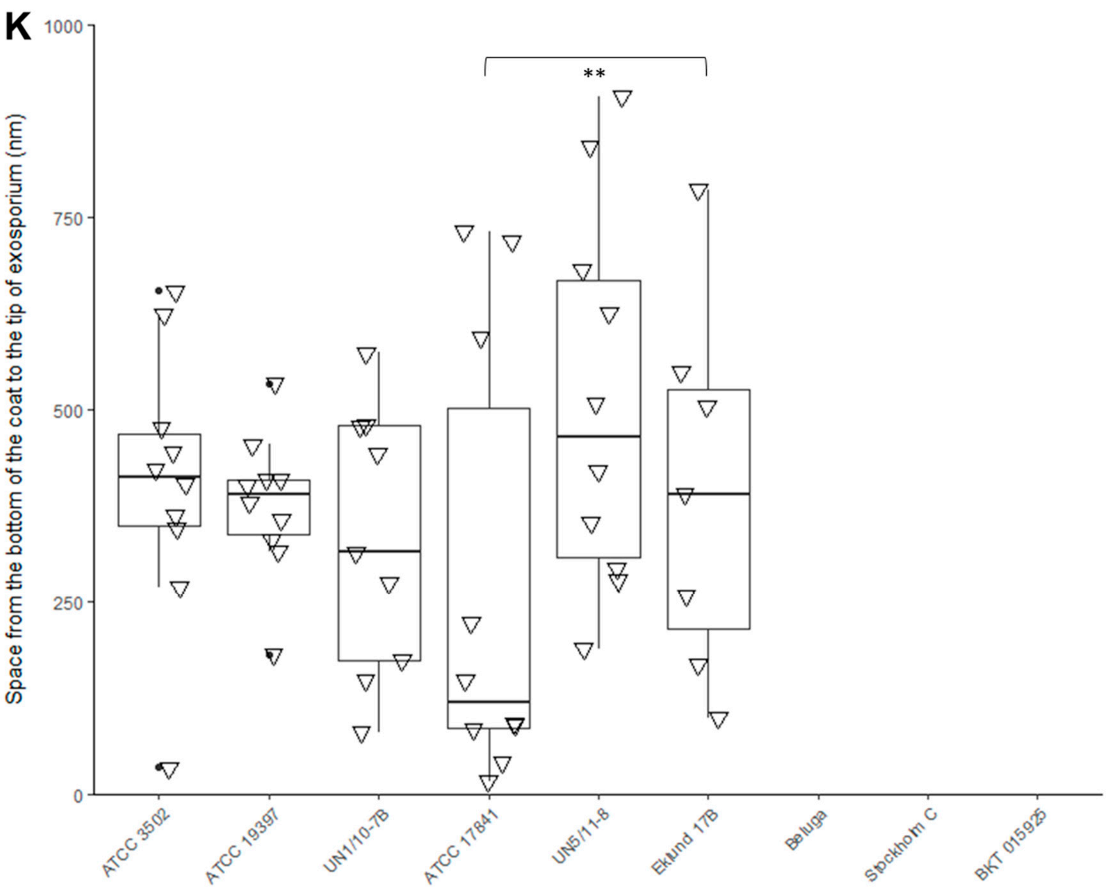
K
Space from the bottom of the coat to the tip of exosporium (nm)
1000
750
500
250
0
**
ATCC 3502
ATCC 19397
UN1/10-7B
ATCC 17841
UN5/11-8
Eklund 17B
Beluga
Stockholm C
BKT 015925

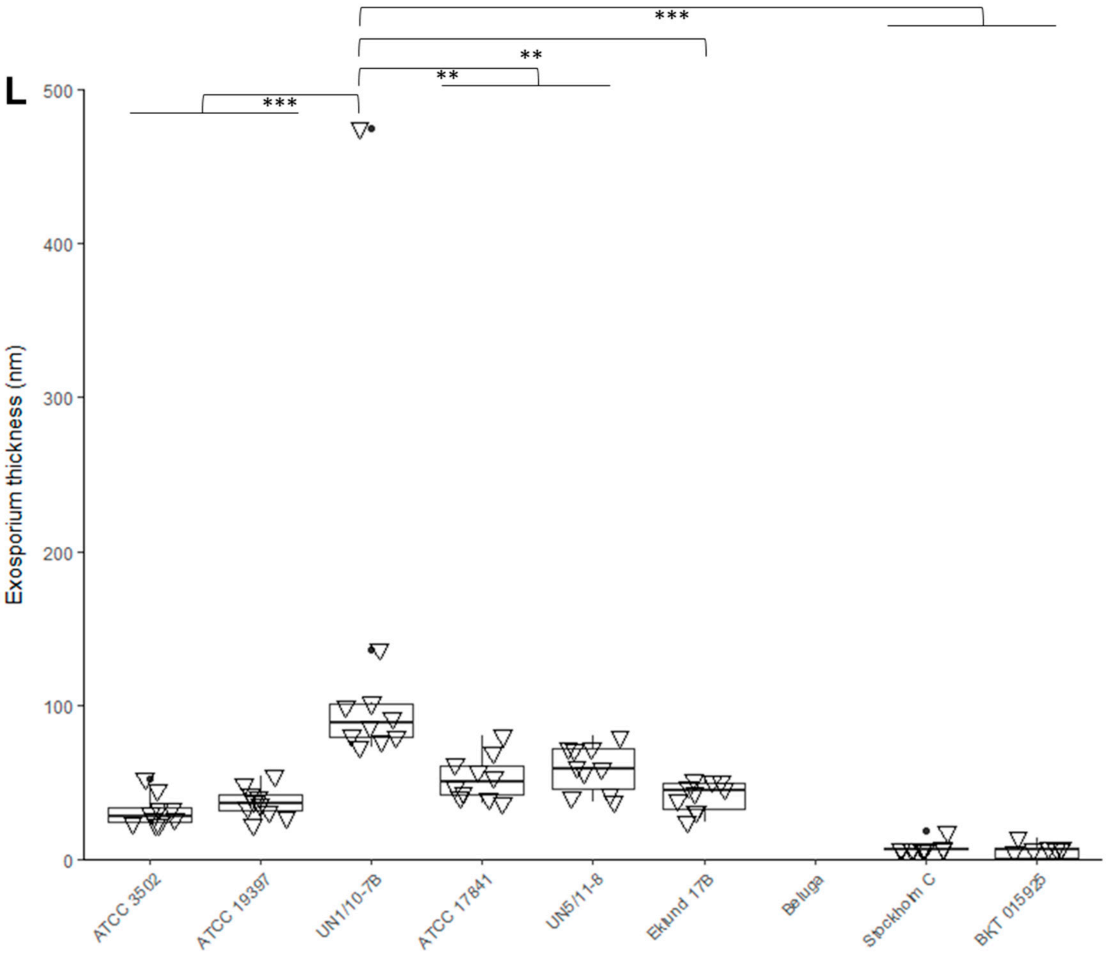
L
Exosporium thickness (nm)
500
400
300
200
100
0
***
**
**
***
ATCC 3502
ATCC 19397
UN1/10-7B
ATCC 17841
UN5/11-8
Eklund 17B
Beluga
Stockholm C
BKT 015925

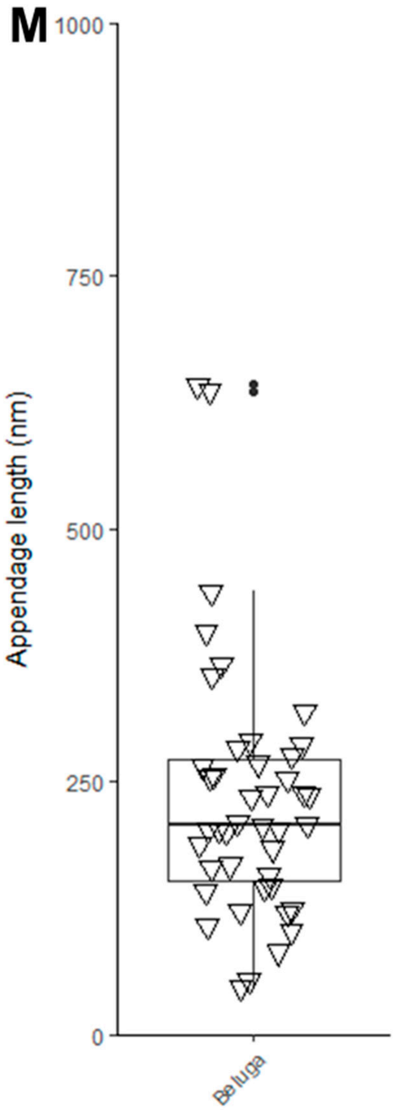

Figure S4. Ultrastructures of purified *Clostridium botulinum* spores. Represented are the measurements and statistical analysis of (**A**) length of the spore with exosporium, (**B**) width of the spore with exosporium, (**C**) length of the spore without exosporium, (**D**) width of the spore without exosporium, (**E**) core length, (**F**) core width, (**G**) cortex thickness, (**H**) coat thickness, (**I**) space between coat and exosporium, (**J**) space from the top of the coat to the tip of exosporium, (**K**) space from the bottom of the coat to the tip of exosporium, (**L**) exosporium thickness, and (**M**) appendage length. Box plots were used to represent the distribution of the measurements, while the individual measurements are represented by triangles. The p-values are represented as *$p < 0.05$, **$p < 0.01$, ***$p < 0.001$.

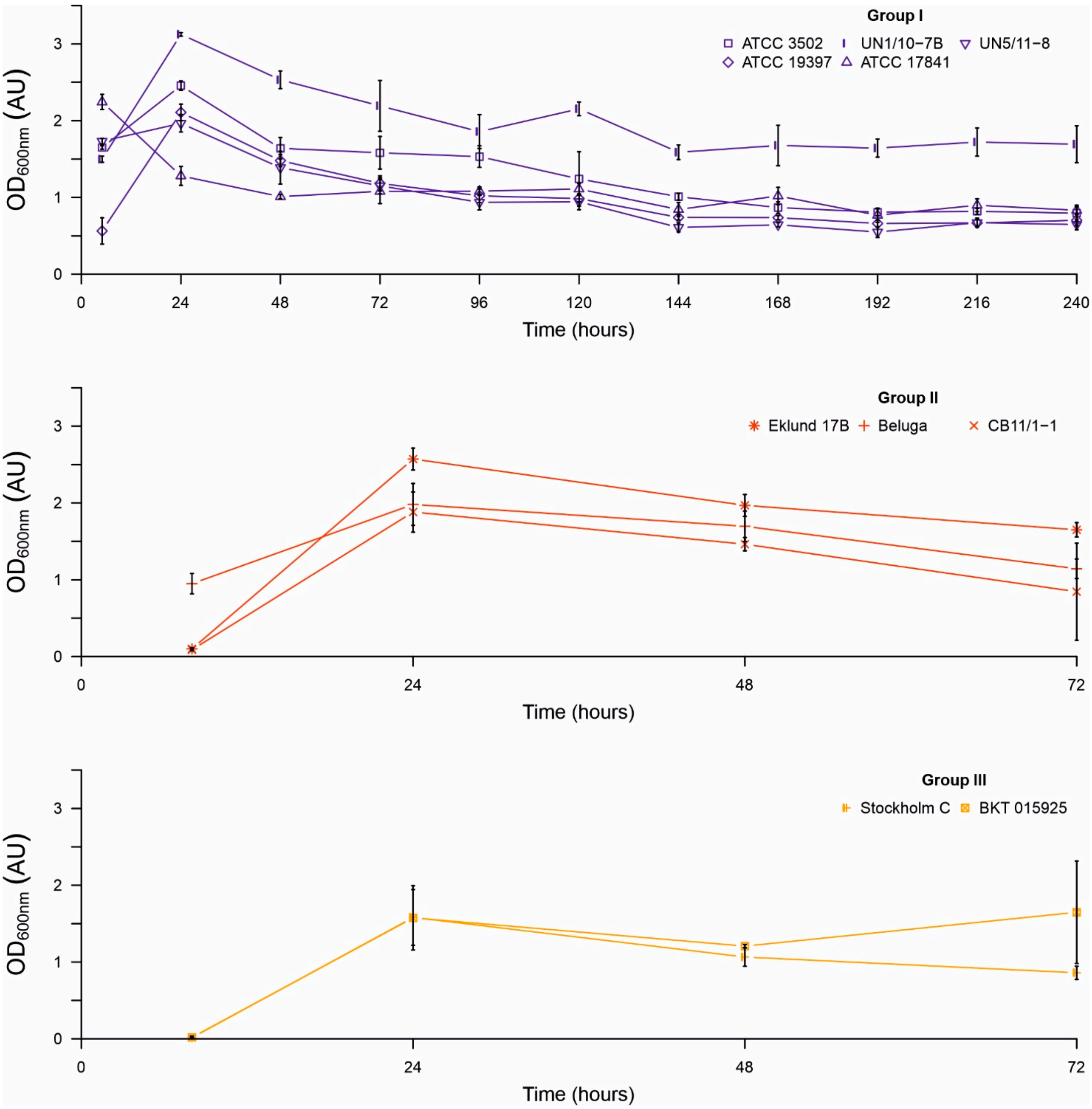

Figure S5. $OD_{600}nm$ of sporulating *Clostridium botulinum* cultures. Values are the average OD_{600nm} of three or two (UN1/10-7B) replicate cultures. Group I strains are represented in blue, Group II in red, and Group III in orange.

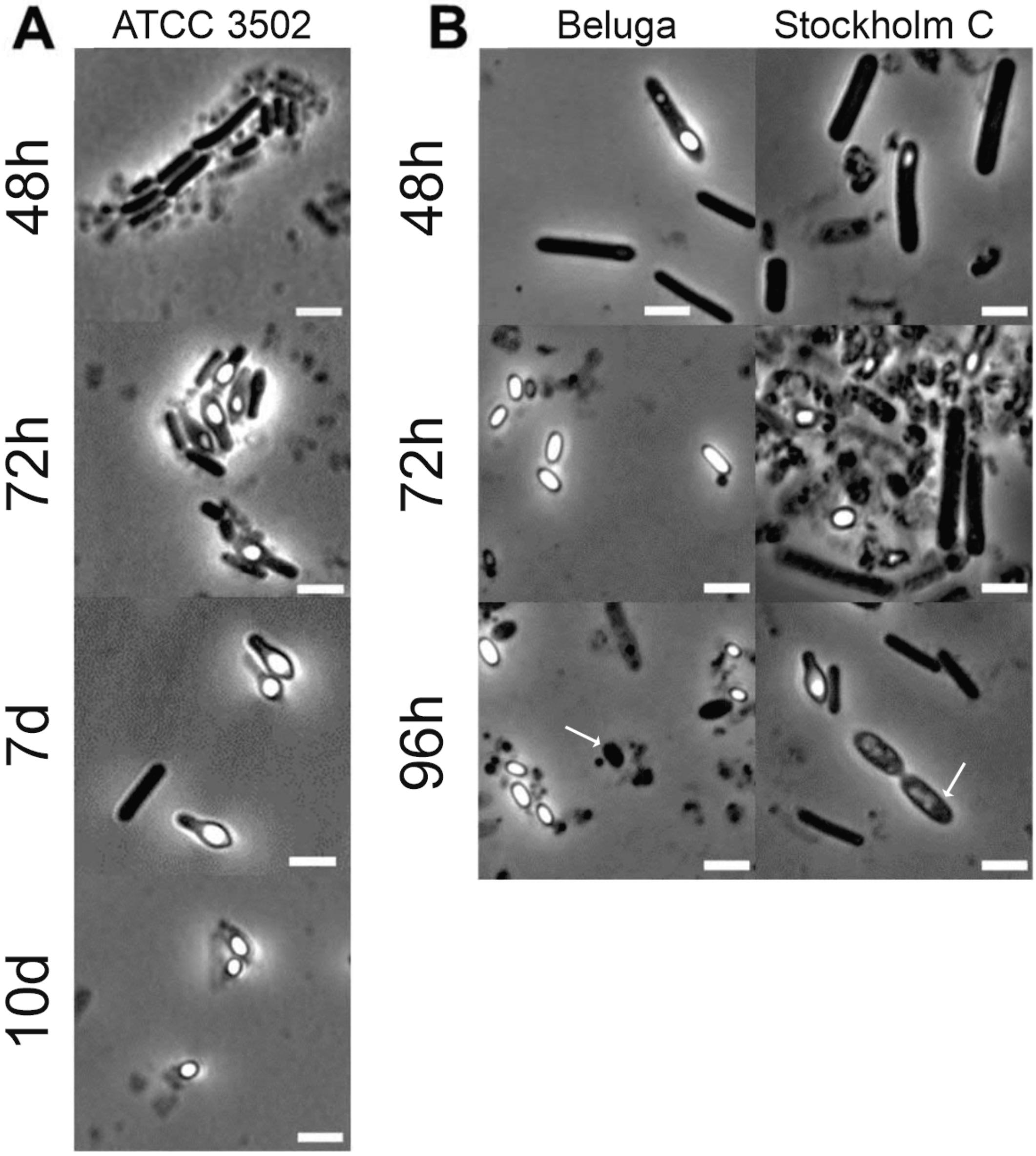

Figure S6. Sporulating cultures of *Clostridium botulinum* (**A**) Group I and (**B**) Group II and III strains. Time of culture collection for spore purification was set aiming to a time where a substantial amount of free spores was present without free phase-dark spores (visible at 96 hours in Beluga and Stockholm C, white arrows): 10 days for Group I strains and 3 days for Groups II and III. Scale bars represent 1 μm.

Table S1. Types and numbers of measurements taken for each of the ten *Clostridium botulinum* spores selected for each strain.

Measurement	Number of measurements per spore
Length spore (with exosporium)	1
Width spore (with exosporium)	1
Length spore (without exosporium)	1
Width spore (without exosporium)	1
Length spore core	1
Width spore core	1
Cortex thickness	3
Coat thickness	3
Space between the coat and exosporium	3
Space from the top of the coat to the tip of exosporium	1
Space from the bottom of the coat to the bottom of exosporium	1
Exosporium thickness	3
Beluga appendages	All present in each spore
